# Diverse Roads Taken by ^13^C-Glucose-Derived Metabolites in Breast Cancer Cells Exposed to Limiting Glucose and Glutamine Conditions

**DOI:** 10.3390/cells8101113

**Published:** 2019-09-20

**Authors:** Maria Gkiouli, Philipp Biechl, Wolfgang Eisenreich, Angela M. Otto

**Affiliations:** 1 Munich School of BioEngineering, Technical University of Munich, 85748 Garching, Germany; mariagkiouli@gmail.com (M.G.); philipp.biechl@tum.de (P.B.); 2 Department of Chemistry, Chair of Biochemistry, Technical University of Munich, 85748 Garching, Germany; wolfgang.eisenreich@mytum.de

**Keywords:** nutrient deprivation, ^13^C-metabolites, isotopologue distribution, glycolysis, Warburg effect, TCA cycle, anaplerosis, gluconeogenesis, breast cancer cells

## Abstract

In cancers, tumor cells are exposed to fluctuating nutrient microenvironments with limiting supplies of glucose and glutamine. While the metabolic program has been related to the expression of oncogenes, only fractional information is available on how variable precarious nutrient concentrations modulate the cellular levels of metabolites and their metabolic pathways. We thus sought to obtain an overview of the metabolic routes taken by ^13^C-glucose-derived metabolites in breast cancer MCF-7 cells growing in combinations of limiting glucose and glutamine concentrations. Isotopologue profiles of key metabolites were obtained by gas chromatography/mass spectrometry (GC/MS). They revealed that in limiting and standard saturating medium conditions, the same metabolic routes were engaged, including glycolysis, gluconeogenesis, as well as the TCA cycle with glutamine and pyruvate anaplerosis. However, the cellular levels of ^13^C-metabolites, for example, serine, alanine, glutamate, malate, and aspartate, were highly sensitive to the available concentrations and the ratios of glucose and glutamine. Notably, intracellular lactate concentrations did not reflect the Warburg effect. Also, isotopologue profiles of ^13^C-serine as well as ^13^C-alanine show that the same glucose-derived metabolites are involved in gluconeogenesis and pyruvate replenishment. Thus, anaplerosis and the bidirectional flow of central metabolic pathways ensure metabolic plasticity for adjusting to precarious nutrient conditions.

## 1. Introduction

The manifestation of a reprogrammed metabolism is a hallmark of cancer cells [1,2], and a better understanding of its dynamics is a central issue for diagnostics, especially those based on metabolic imaging, for antimetabolite therapies, as well as for dietary cancer prevention. The Warburg effect, that is, high lactate production in aerobic conditions, is considered to be the common metabolic feature of tumor cells [3,4]. However, even within the same tumor, there are subpopulations of cells that may differ in their metabolic activities depending not only on their genetic disposition, but also on their microenvironment. The latter is characterized by fluctuating levels of soluble components, such as growth factors and nutrients, which depend on the diffusion properties of the interstitial space and the distance from a blood vessel. Glucose and glutamine are the key substrates for maintaining both anabolic and catabolic metabolism, and thus cell growth [5]. Physiological plasma levels of glucose are in the range between 5 to 7 mM [6], and those of glutamine are at about 0.6 mM [7,8]. However, in normal tissues and especially in solid tumors with poor vasculature, glucose has been reported as being undetectable in tumor interstitium [9] or well below 4 mM in tumor tissues [10,11,12], depending on the heterogeneity of the tumor. Moreover, the concentration of arterial glutamine in cancer tissue falls well below 0.5 mM [13]. Therefore, tumor cells are exposed to an increasingly precarious microenvironment to which they must adapt their metabolism efficiently to meet the demands for sustaining their survival.

There is a paucity of studies on the metabolic network in conditions of nutrient deprivation mimicking the microenvironment of cancers; in most studies, cultures were deprived of *either* glucose *or* glutamine. Such deprivation may differentially affect tumor cells depending on their status of mutated or deleted oncogenes and genes for transporters and metabolic enzymes [14]. For example, silencing the tumor suppressor gene CC3 in HeLa cells allowed them to survive longer in low glucose than in saturating conditions [15]. In the malignant, K-ras-activated breast cancer cells MDA-MB231, low glutamine with high glucose diminished the growth rate, while conversely, low glucose in the presence of high (4 mM) glutamine virtually stopped it [16]. Moreover, as shown for the low-malignant myc-expressing breast cancer cell line MCF-7, limiting glucose and glutamine levels modifies cell growth as well as the activities of pyruvate kinase, lactate dehydrogenase (LDH), and plasma membrane NADH-oxidase, depending on the glucose/glutamine ratio [17]. It is, therefore, prudent to get a better understanding of tumor metabolism in various precarious nutrient conditions.

Metabolomic technologies using gas chromatography in conjunction with mass spectrometry (GC/MS) or liquid chromatography (LC/MS) and stable isotope (e.g., ^13^C) tracking provide an increasingly complex picture of metabolism by discerning the interplay of different metabolic pathways, such as glycolysis, the TCA cycle, and anaplerosis by glutamine and pyruvate [16,18,19]. Such studies have revealed metabolic heterogeneity in lung cancers, showing that cancer cells had a higher lactate metabolism than benign and non-cancerous cells, and this was associated with pyruvate anaplerosis [20,21,22]. The role of pyruvate carboxylation was particularly evidenced in metastatic breast cancer cells [23], its engagement being higher at the site of lung metastasis than at the primary site [24]. Moreover, in lung cancers, upon glucose depletion, ^13^C-lactate carbons were found in ^13^C-phosphoenolpyruvate, indicating gluconeogenesis [25]. These reports illustrate how the interplay of different metabolic pathways reflects and affects the oncogenic behavior. In spite of its fundamental interest, there is no systematic analysis of how limiting glucose and glutamine levels modulate these different metabolic pathways.

The objective of this study, therefore, was to get a more comprehensive and unbiased overview of the metabolic pathways in a breast cancer cell line by concomitantly limiting both glucose and glutamine levels, based on data from a previous study with MCF-7 cells [17]. This cell line has served as a model system in numerous studies on growth control and genomics, for drug screening, and for xenographs in mice [26], albeit generally in high glucose and glutamine conditions (11–25 mM, 4 mM, respectively). To reduce the intrinsic heterogeneity of a three-dimensional tissue, these epithelial-like cells were cultivated as monolayers, in which all cells are exposed to the same medium conditions. After an adaptive period to limiting glucose (1 mM; 2.5 mM) and glutamine (0.1 mM; 1 mM) conditions to mimic precarious nutrient availability, these cells were incubated with the respective concentrations of [U-^13^C_6_]glucose. Considering that the extracellular milieu could change during the incubation, as may occur during the growth of a solid tumor lacking ample blood supply, this approach does not *a priori* assume steady-state conditions. For this reason, we used an observation-driven approach by comparing ^13^C-enrichments and isotopologue distribution in key metabolites at 2 and 20 h of [U-^13^C_6_]glucose incubation in media with different glucose and glutamine combinations. Our data show that (1) total as well as ^13^C-labeled metabolite pools change with the different nutrient conditions; (2) ^13^C-glucose-derived metabolites were variably engaged in glycolysis and the oxidative TCA cycle, including pyruvate and glutamine anaplerosis, as well as gluconeogenesis; and (3) limiting glucose and glutamine conditions lead to a modulation in metabolic fluxes, including lactate release, that is, the Warburg effect. These results illustrate the high metabolic plasticity of tumor cells in a fluctuating low nutrient microenvironment.

## 2. Materials and Method

### 2.1. Cell Culture

Breast adenocarcinoma (MCF-7) cells were originally obtained from the Institut Jules Bordet, (Brussels, Belgium), and their identity was confirmed in August 2015 by the Leibniz-Institut DMSZ GmbH, Braunschweig, Germany. The cells were routinely maintained in standard Dulbecco’s Modified Eagle Medium (DMEM; Sigma-Aldrich, Taufkirchen, Germany) containing 4.5 g/L (25 mM) glucose, 0.58 g/L (4 mM) glutamine, and 5% fetal calf serum (FCS; Biochrom, Berlin, Germany), but no antibiotics, at 37 °C with 10% CO_2_. Cells were free of mycoplasma as indicated by fluorescent DNA staining. For limiting nutrient conditions, the culture media consisted of DMEM-base (without glucose, glutamine, NaHCO_3_, phenol red; Sigma-Aldrich, Taufkirchen, Germany) supplemented with 2% FCS, 50 nM insulin, 3.7 g NaHCO_3_/L for a pH of 7.4, and combinations of glucose (1, 2.5, 25 mM) and glutamine (0.1, 1.0, 4.0 mM) as indicated in the experiments and published before [17]. Note: DMEM contains 0.4 mM serine and 0.4 mM glycine, but no alanine.

### 2.2. Cell Cultures and Preparation of Cell Lysates for Biochemical Analyses

Cell were seeded in six-well plates at 4 × 10^4^ cells/2 mL standard medium, which was exchanged for medium with the different glucose and glutamine conditions in triplicates after 48 h. After a subsequent three-day incubation, media were exchanged with respective fresh medium, and cells were incubated for 2 and 20 h, as in the protocols for the ^13^C-labeling experiments. Supernatants were then collected and ice-cold hypotonic buffer (20 mM HEPES, 0.5 mM CaCl_2_, 1 mM MgCl_2_) was added to the monolayer for 20 min. After removal of the buffer, cells were suspended by scraping them from the dish, and transferred to a pre-cooled tube, with the final volume adjusted to 200 µL with the hypotonic buffer.

### 2.3. Cell Number Determination

Cell number was determined by nuclei counting, basically as described before [17]. With the ^13^C-labeled cultures, an aliquot of trypsinized cells was centrifuged, the pellet suspended in ice-cold hypotonic buffer (as above) and left at 6–8 °C for 20 min before adding 5% benzalkonium (Sigma-Aldrich) in acetic acid. The resulting nuclei suspensions were counted in an electronic cell counter (Casy, OLS, Bremen, Germany).

### 2.4. Glucose Determination

Glucose remaining in the cell culture supernatants and cell lysates was determined using the Amplex^®^ Red Glucose/Glucose Oxidase Assay Kit (Invitrogen, Life Technologies). Fluorescence was measured after 30 min at the excitation and emission wavelengths of 530 and 590 nm, respectively (multimode microplate reader Victor 3, PerkinElmer, Waltham, MA, USA). Glucose concentrations were calculated from a standard glucose curve set up for each experiment.

### 2.5. Lactate Determinations of Cells and Culture Supernatants

Lactate content in cell lysates and lactate released into the culture medium were measured using the NADH optical test according the reference [27]. For determining the intracellular lactate levels, 100 µL of a lysate from 1 × 10^6^ trypsinized cells, prepared with hypotonic buffer indicated above, was precipitated by adding 33.3 µL 4 M perchloric acid on ice for 20 min. Following centrifugation at 13,000× *g*, 100 µL of supernatant was collected and adjusted to pH 6.5–8.5 by adding 64.6 µL of ice-cold 2 M KOH for 20 min. The resulting precipitate was centrifuged and the supernatant was collected for lactate determination. An aliquot of supernatant and lysate samples was added to a buffer containing 1.1 M hydrazine (pH 9.0), 5 mM NAD+, and L-LDH (rabbit muscle, approximately 1000 units/mL, Sigma). After incubating for 30 min at room temperature, the change in fluorescence was measured at the excitation and emission wavelengths of 355 and 460 nm, respectively (Victor 3, Perkin Elmer). Lactate concentrations were calculated from a reference curve. The intracellular lactate concentration was calculated based on the cell number of the lysate and a volume of 2 × 10^−12^ L per cell, as determined by microscopy and the size of distribution given by the Casy counter.

### 2.6. Determination of Enzyme Activities

For measuring pyruvate kinase activity, cell lysates were added to a buffer containing L-LDH, phosphoenolpyruvate (PEP), and ADP. LDH activity was determined with cell lysates added to a buffer containing pyruvate as substrate with NADH for the forward reaction (LDH-A activity). Linear changes in NADH absorption were monitored for <1 min at 340 nm (Specord 210, Analytik Jena AG, Jena, Germany), as described before [17]. Specific enzyme activity was expressed as units (µmol/min) per 10^6^ cells. It should be noted that the enzyme assays measure the potential activity with a saturating substrate concentration, not necessarily representing actual cellular concentrations in the different nutrient conditions.

### 2.7. ^13^-C-Labeling Experiments

Cells were seeded at 6 × 10^5^ cells in 30 mL standard medium in 15 cm cell culture Petri dishes and incubated for three days. The medium was then exchanged for media with the glucose and glutamine composition, as indicated in the experiments. These cultures were incubated for three additional days. Then, the cultures received fresh media with the same compositions, but with glucose replaced by the same concentrations of [U-^13^C_6_]glucose, and were incubated for another 2 and 20 h. Cell culture supernatants were then collected for determination of glucose and lactate levels. The adherent cells were washed with phosphate-buffered saline (PBS), trypsinized, centrifuged, and resuspended in ice-cold PBS, of which an aliquot was taken for cell counting. The main suspension was centrifuged again and flash-frozen in liquid nitrogen (LN_2_). Frozen cell pellets were subsequently lyophilized for 24 h, weighed, and stored at −20° C until GC/MS analysis.

### 2.8. Extraction of Polar Metabolites

A mixture was prepared containing <10 mg of a lyophilized sample from the ^13^C-labeled cells, suspended in 1 mL methanol (100%), doped with 0.4 mM norvaline as an internal standard, and 500 μL glass beads. This mixture was transferred to a ribolyser tube, and cells were disrupted mechanically five times for 5 s at 6.5 m/s. The sample was then centrifuged for 10 min at 5000× *g* at 4 °C, and the resulting supernatant, containing the polar metabolites, was transferred to a glass vial and blow-dried with nitrogen. For derivatization of keto and aldehyde groups, the sample was incubated with 100 μL of methoxyamine hydrochloride in pyridine (20 mg/mL) for 90 min at 40 °C and blow-dried with nitrogen at 40 °C. Finally, the sample was silylated in 100 μL of N-trimethylsilyl trifluoroacetamide (MSTFA) for 30 min at 45 °C. All samples were analyzed by GC/MS using a quadrupol GCMS-QP 2010 Plus spectrometer with Auto injector AOC-20i (Shimadzu, Duisburg, Germany) and a slightly modified protocol, as described before in detail [28]. The GC column was a silica capillary column (equity TM-5; 30 m by 0.25 mm, 0.25-µm film thickness; Sigma-Aldrich, Taufkirchen, Germany). For the analysis of the silylated metabolites, the column was developed at 70 °C for 5 min followed by a gradient (3 °C per min) up to 210 °C. At the end, the column was quickly heated to 310 °C (20 °C per min) and further developed at this temperature for 2 min. Each sample was measured three times with average deviations of <3%.

### 2.9. Data Analysis

The obtained GC/MS data were evaluated with the software “GCMSsolution” from Shimadzu. Overall ^13^C-enrichments and isotopologue compositions were calculated by comparing with unlabeled samples using Excel scripts and according to Ahmed et al. [29]. This software package is open-source and can be downloaded by the link: http://www.tr34.uni-wuerzburg.de/software_developments/isotopo/. All ^13^C-enrichment values were corrected for 1.1% of ^13^C-natural abundance. Each ^13^C-labeling experiment in the different growth conditions was performed twice, except for the combination 0.1 mM glutamine/1 mM glucose, which was done four times. The figures show the averages of these experiments, with the error bars denoting the range of individual data points.

## 3. Results

### 3.1. Changes in the Warburg Effect in Limiting Nutrient Conditions

To mimic growth in limiting nutrient conditions, cells were cultured in media containing glucose (1 and 2.5 mM) and glutamine (0.1 and 1 mM) in various combinations. For comparison with the standard, that is, saturating, cultivation conditions, cells were also cultured in medium containing 25 mM glucose and 4 mM glutamine. After three days, the cellular status was characterized by various parameters before (t = 0 h) and, in accordance with the ^13^C-glucose labeling protocol, at 2 and 20 h after changing the medium with the same composition.

#### 3.1.1. Cell Growth

**Figure 1:**
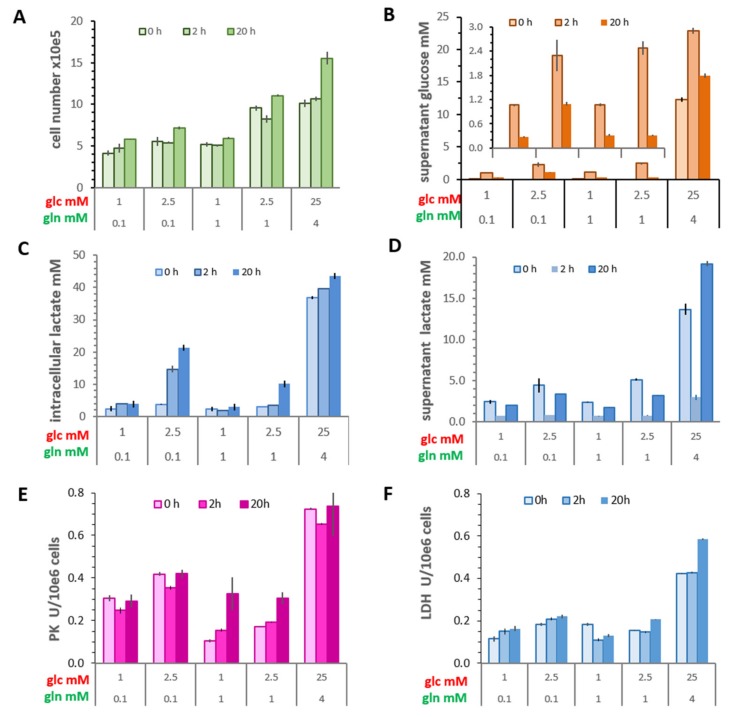
Metabolic characterization of MCF-7 cells after incubating for three days (t = 0) and then 2 and 20 h following the replenishment with the same medium composition as indicated by the concentrations (mM) of glucose and glutamine. Cells were cultured in six-well plates as described in Material and Methods. (**A**) Cell number. (**B**) Glucose concentration remaining in the culture medium (2 mL). The insert enlarges the small values in limiting medium conditions. (**C**) Intracellular lactate concentrations. (**D**) Lactate concentration in culture supernatants (2 mL). Potential cellular activities of (**E**) pyruvate kinase (PK) and (**F**) lactate dehydrogenase (LDH). Shown are the averages of triplicate cultures with their deviations.

A three-day incubation period was chosen based on a previous study [17], which showed a reduction in cell numbers with prolonged incubation owing to cell death. Therefore, cells were incubated no longer than 72 h. In the present experiments (Figure 1A), cell numbers in the most limiting glucose and glutamine conditions (1 mM glucose/0.1 mM glutamine) were about 50% lower than in standard culture conditions, reflecting differences in cell proliferation and increased cell death [17]. Upon medium change with the same compositions, a substantial increase in cell number was observed only after 20 h, mainly in the standard conditions.

#### 3.1.2. Glucose Consumption

In standard medium initially containing 25 mM of glucose, cells had consumed about half of the available glucose within three days (Figure 1B). In contrast, little or no measurable glucose remained in cultures with low glucose conditions, similar to results reported previously [17]. Upon replacing the conditioned medium with medium containing the respective glucose/glutamine concentrations, only a marginal loss of glucose was measurable at 2 h. However, after 20 h, up to 90% of the glucose had been consumed in media with 2.5 mM glucose/1 mM glutamine, while it was only about 55% in cultures with initially 2.5 mM glucose/0.1 mM glutamine, a difference not simply accounted for by differences in cell number. Actually, cells growing in 2.5 mM glucose apparently have a higher initial consumption rate with 0.1 mM than with 1 mM glutamine (Figure S1). This indicates that in limiting conditions, the level of glutamine is critical for glucose consumption. Not surprisingly, cells in saturating medium had taken up only about 30% of the initial glucose.

#### 3.1.3. Intracellular Lactate Concentrations and Lactate Released

The original experimental set-up of Warburg and Minami [3], like many similar thereafter, measured only extracellular lactate levels. We now asked how these correlated with the level of intracellular lactate. After three days in saturating conditions, the lactate released into 2 mL supernatant amounted to about 28 micromoles, resulting in a concentration of 14 mM, a value also observed in different tumors [12] (Figure 1D). The total lactate in the cells of this culture was about 93 nanomoles, which, when calculated per cell volume (2 pL), amounted to an intracellular lactate concentration of about 37 mM (Figure 1C). After three days in the limiting conditions, the amount of intracellular lactate was dependent on the glucose/glutamine combination, ranging between a mere 1.7 up to 5 nanomoles per culture, thus resulting in intracellular concentrations between 2.3 and 3.7 mM. Here, the supernatant lactate concentrations were 5–10 mM. It is noteworthy that after 2 and 20 h of medium replenishment with 2.5. mM glucose/0.1 mM glutamine, the increase in the intracellular lactate was up to 20 mM, that is, about 10-fold higher than in the other limiting conditions. Yet, the extracellular lactate level remained below 5 mM, even at 20 h, thus sustaining a substantial intra-/extracellular lactate concentration gradient. These numbers show that cells can maintain a relatively constant intracellular concentration of lactate over time, which is only partially dependent on extracellular glucose levels, while lactate accumulation in the medium is dependent on the growth conditions, resulting mainly from glycolytic activity. Thus, the amount of lactate released, namely the Warburg effect, did not reflect the intracellular lactate content.

#### 3.1.4. Activities of Pyruvate Kinase and LDH

After a three-day incubation in limiting glucose conditions, the potentially available activity of pyruvate kinase was lower in cells with 1 mM than with 0.1 mM glutamine (Figure 1E), corresponding with glucose depletion. Only cells in saturating medium and in medium with 2.5 mM glucose/0.1 mM glutamine maintained higher enzyme activities (0.8 units and 0.4 units/10^6^ cells, respectively), indicating a regulatory interplay of glucose and glutamine [17]. This activity was only slightly changed 2 h after glucose replenishment, implying no changes in the level of the enzyme proteins. At 20 h, only those cells maintained in 1 mM glutamine in combination with low glucose showed an increase in pyruvate kinase activity, suggestive of an increased glycolytic rate. In contrast, the activity of LDH showed less changes with the different conditions and time points. Moreover, in MCF-7 cells, the LDH-A activity (0.2 units/10^6^ cells) was consistently lower than of that of pyruvate kinase, and a 2–3-fold higher value was obtained only for cells in saturating growth medium (Figure 1F). In the limiting conditions, neither glucose consumption nor lactate accumulation in the medium had a measurable effect on the potential LDH-A activity.

### 3.2. Changes in the Levels of Intracellular Metabolites

**Figure 2:**
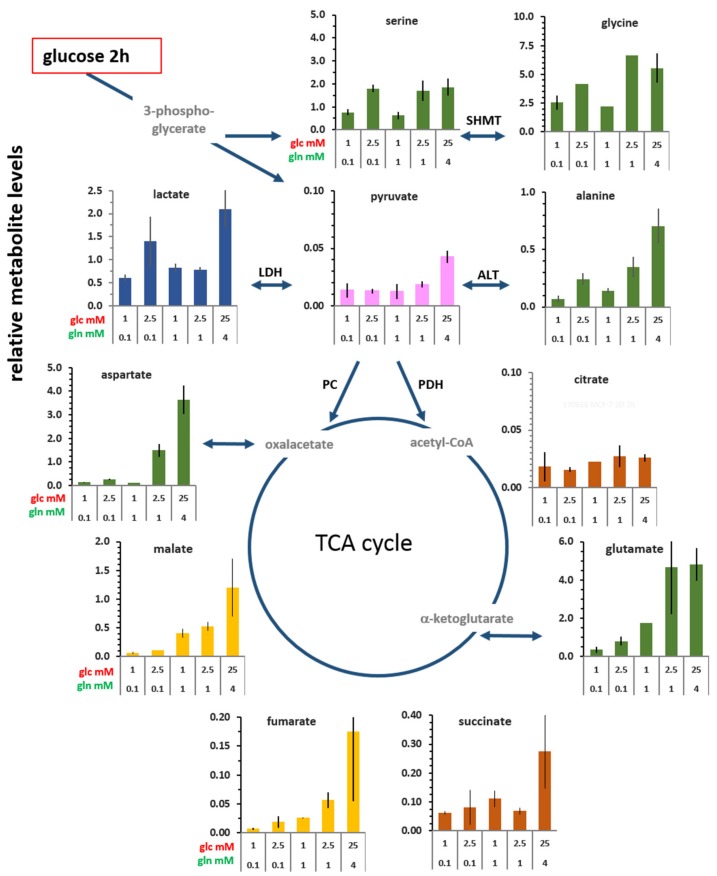
Cellular metabolite levels 2 h after medium replenishment with various glucose/glutamine combinations. The metabolites detected by gas chromatography/mass spectrometry (GC/MS) were quantified with reference to norvaline (=1) and expressed as relative values. The data of these metabolites for cells at 2 and 20 h are compiled in Figure S2, and the graphs for 20 h are presented in Figure S3. Shown are the averages and the range of the data points of individual experiments. SHMT: serine hydroxymethyl transferase; LDH: lactate dehydrogenase; ALT: alanine aminotransferase; PC: pyruvate carboxylase; PDH: pyruvate dehydrogenase. Oxaloacetate, acetyl-CoA, and α-ketoglutarate could not be detected.

The cellular levels of glucose-derived metabolites were determined after 2 and 20 h incubations with [U-^13^C_6_]glucose in combination with different glutamine concentrations (Figure 2 and Figures S1 and S2). Owing to the necessity of chemical derivatization of the soluble polar metabolites for detection by GC/MS, metabolite levels were quantified relative to the derivatized reference compound, norvaline (see Material and Methods). Intracellular glucose was not identifiable with this GC/MS protocol. Also, using the same glucosidase assay as for glucose determination in the culture medium, glucose was not reliably detectable in cell lysates, even in standard conditions (data not shown). Possibly, most of the free glucose is rapidly converted to glucose-6-P for glycolysis.

Even though there are differences in the efficiencies of metabolite derivatization, it is safe to say that the lowest metabolite levels were found for pyruvate and citrate, their lack of substantial accumulation suggesting a transitory role in metabolic flux. Technical limitations did not allow for the detection of α-ketoglutarate and oxaloacetate, so the respective amino acids, glutamate and aspartate, may serve as their tentative proxies. The metabolite levels were essentially similar at 2 and 20 h (Supplementary Figure S2), implying that cells maintained a rather stable (pseudo-steady state) level of these central metabolites over time, despite the fact that glucose levels were reduced after 20 h.

The levels of each metabolite were generally reduced in limiting glucose and glutamine conditions, compared with standard conditions. Of the glycolysis-associated metabolites, the levels of serine and glycine (synthesized via 3-phosphoglycerate) in cells with 2.5 mM glucose were very similar to those in standard medium; yet at 1 mM glucose, their levels were 50% lower (Figure 2). This indicates that intracellular levels of serine and glycine are more glucose than glutamine dependent. Limiting glucose availability of 1 mM and 2.5 mM also reduced the levels of pyruvate and lactate by about two-fold relative to conditions with 25 mM glucose. However, in cells with 2.5 mM glucose/0.1 mM glutamine, lactate content was enhanced (Figure 2; also at 20 h, Figure S3), in line with the enzymatic lactate determination as well as with increased activity of pyruvate kinase (Figure 1C,E). In contrast, alanine levels were sensitive to both the concentration as well as the ratio of glucose/glutamine (Figure 2). Thus, changes in alanine levels did not parallel those of pyruvate or lactate levels in the different glucose/glutamine combinations, indicating different regulations of pyruvate utilization.

Of the TCA cycle metabolites at 2 h, citrate showed little nutrient dependent variations (Figure 2). Also, the levels of succinate varied little at limiting conditions, except for a 4.5-fold enhancement in standard conditions. In contrast, the levels of glutamate, fumarate, and malate increased with increasing glucose and glutamine concentrations, being markedly high with 25 mM glucose/4 mM glutamine. The differences between the lowest and highest levels were about 25-fold for fumarate, 18-fold for malate, and 30-fold for aspartate (proxy for oxaloacetate), and their levels were highly glutamine dependent, indicative of glutamine anaplerosis.

### 3.3. ^13^C-enrichment of Glucose-Derived Metabolites in Limiting Nutrient Conditions

**Figure 3:**
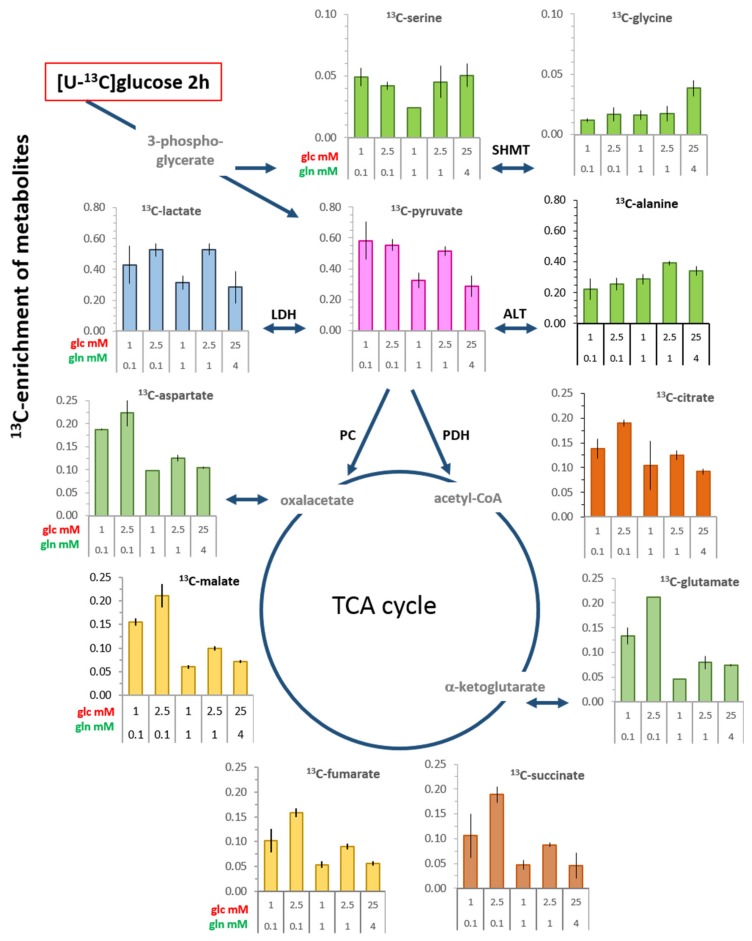
^13^C-enrichment of metabolites from cells incubated for 2 h with [U-^13^C_6_]glucose at the indicated glucose and glutamine concentrations. Cells were extracted and ^13^C-metabolites were analyzed as described in Material and Methods. ^13^C-enrichment is expressed as the labeled fraction in the metabolite pool, as depicted in Supplementary Figure S7B. Shown are the averages and the range of the data points of individual experiments. Graphs of cells at 20 h are presented in Supplementary Figure S4.

^13^C-enrichments in key metabolites were determined following the incorporation of [U-^13^C_6_]glucose for 2 h (Figure 3) and 20 h (Supplementary Figure S4). The focus here will be on the 2 h time point as the one more closely representing the metabolic changes of the cells following medium replenishment than after 20 h, when the medium composition will have changed, for example, by glucose consumption and lactate accumulation (Figure 1B,D).

#### 3.3.1. Glycolysis-Associated Metabolites

^13^C-labeled intermediates along the glycolytic pathway preceding pyruvate were below the detection level. However, ^13^C-enrichments of serine and glycine, as metabolites derived from ^13^C-3-phosphoglycerate, were measurable after 2 h, for serine being between 5% and 10% and for glycine being less than 5% (Figure 3). For serine, the lowest ^13^C-enrichment was observed in the condition with 1 mM glucose/1 mM glutamine, a physiologically unbalanced combination that has been shown to attenuate cell survival [17]. After 20 h, the ^13^C-levels of both metabolites had increased almost two-fold in a glucose-dependent manner (Supplementary Figure S4).

High values of ^13^C-enrichment were obtained for ^13^C-pyruvate, being up to 60% in limiting conditions with low (0.1 mM) glutamine in combination with either 1 or 2.5 mM glucose, as well as with 1 mM glutamine/2.5 mM glucose (Figure 3). Lactate showed virtually the same level of ^13^C-enrichment as pyruvate, usually being lower only in the most limiting conditions. On the whole, this indicated a high conversion rate of ^13^C-pyruvate to ^13^C-lactate. In the unbalanced growth condition of 1 mM glucose/1 mM glutamine, ^13^C-enrichment of pyruvate and lactate was reduced by about 45%, which is coincident with reduced pyruvate kinase activity (Figure 1E) and suggests attenuated glycolysis. However, besides the potentially available activity of this enzyme, the level of unlabeled substrates contributes to the ^13^C-enrichment of glucose-derived metabolites. Thus, reduced ^13^C-enrichment was also observed in saturating standard conditions, where it would have been expected that cells growing with 25 mM [U-^13^C_6_]glucose would have the highest ^13^C-enrichment. This not being the case suggests, moreover, that glucose uptake was limited by the K_M_-values of the transporters GLUT1 and GLUT4, which are in the range of 3–5 mM [30]. Additionally, in standard culture conditions, cells may already have had a sufficiently high unlabeled intracellular pool, which further curtailed the uptake and utilization of ^13^C-glucose. This latter argument is supported by data with cells in standard medium, which had higher levels of most metabolites compared with those in limiting conditions (Figure 1 and Figure 2; Supplementary Figure S2). In spite of alanine being a direct transamination product of pyruvate, its ^13^C-enrichment was lower than that of lactate, being in the range of 20%–40%, depending on both glucose and glutamine concentrations (Figure 3). This suggests that not only glucose-derived pyruvate is a precursor for alanine.

#### 3.3.2. TCA Cycle-Associated Metabolites

Of the measured metabolites directly or indirectly related to the TCA cycle, the highest ^13^C-enrichment was about 20% (Figure 3), which was observed with the combination of 2.5 mM glucose/0.1. mM glutamine. Low ^13^C-enrichments were observed in the unbalanced conditions of 1 mM glutamine/1 mM glucose, with values reduced to 5% for glutamate, succinate, fumarate, and malate. There was also a substantial reduction to around 8% in conditions with physiologically saturating 1 and 4 mM glutamine, which is an indication for glutamine anaplerosis [31]. Moreover, a noticeable glutamine-dependent reduction in the enrichment of ^13^C-citrate, similar to that of glutamate, indicates reductive carboxylation of α-ketoglutarate [32]. After a 20 h incubation, all ^13^C-enrichment values had increased, but still showed similar glucose and glutamine dependencies as at 2 h (Supplementary Figure S4).

### 3.4. Changes in Levels of Intracellular ^13^C-metabolites

**Figure 4:**
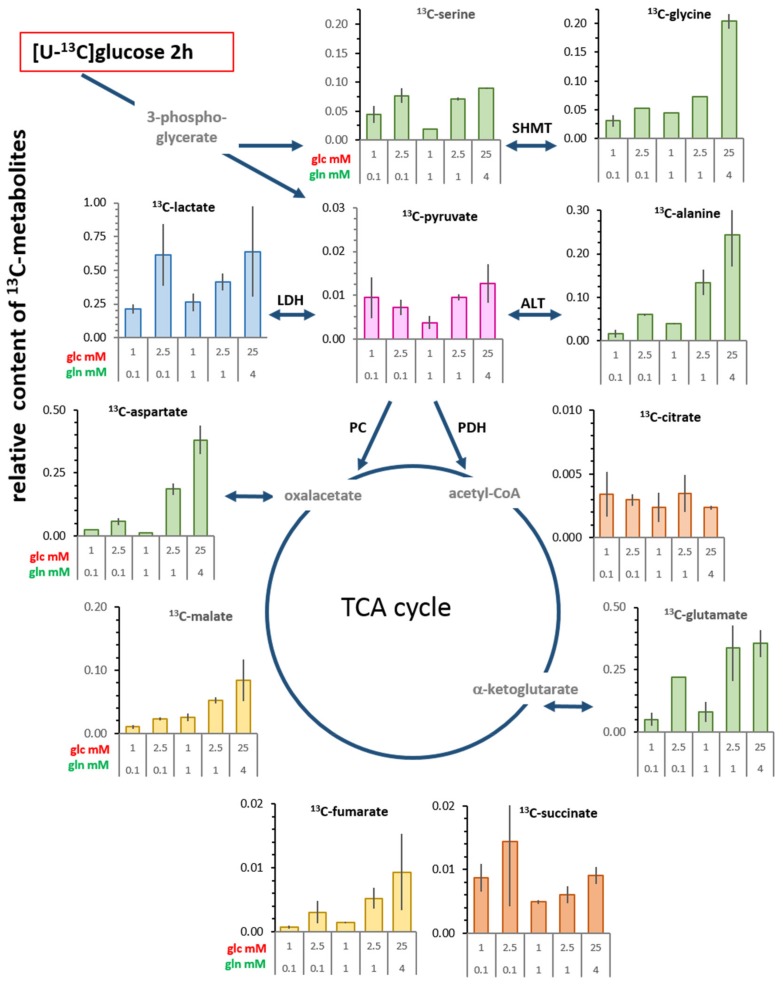
Estimated cellular content of *de novo* ^13^C-glucose-derived metabolites after a 2 h incubation with [U-^13^C_6_]glucose. These values (compiled in Figure S5) give an indication of how different combinations of glucose and glutamine evoke different levels of *de novo* metabolites. The numbers were calculated by multiplying the ^13^C-enrichment value of each metabolite at the indicated glucose and glutamine concentrations (in Figure 3) with the total level of the corresponding metabolite as determined by GC/MS (values in Figure S2). Shown are the averages and the range of calculated values from individual experiments. An informational scheme on the meaning of these values is depicted the Supplementary Figure S7C. The corresponding graphs for 20 h are presented in Figure S6.

A semi-quantitative estimate of the ^13^C-metabolite content illustrates the metabolic contribution of *de novo* metabolites derived from glucose in the cells at 2 h (Figure 4; Supplementary Figure S5) and at 20 h (Supplementary Figures S5 and S6). The most abundant ^13^C-metabolite in all conditions was ^13^C-lactate, its level depending more prominently on the availability of glucose than did pyruvate. The ratio of ^13^C-lactate to ^13^C-pyruvate levels varied markedly with the different conditions, for example, it was about four-fold lower in the most limiting condition than in the saturating conditions or with 0.1 mM glutamine/2.5 mM glucose (Figure 4). This suggests that in suboptimal conditions less pyruvate was converted to lactate. In contrast to other ^13^C-metabolites at 2 h, ^13^C-citrate and ^13^C-succinate contents showed marginal differences between limiting and saturating conditions. Moreover, ^13^C-glutamate and ^13^C-aspartate as well as ^13^C-alanine content were also highly variable; up to 15-fold between limiting and standard glucose/glutamine conditions (Supplementary Data in Figure S5). The cellular content of ^13^C-serine and ^13^C-glycine in limiting conditions (Figure 4; Supplementary Figure S6) showed less variability (maximally four-fold) in the limiting conditions, which may be explained by their extracellular availability.

### 3.5. Kinetics of ^13^C-Enrichment and Evidence for Metabolic Reaction Compartments

**Figure 5:**
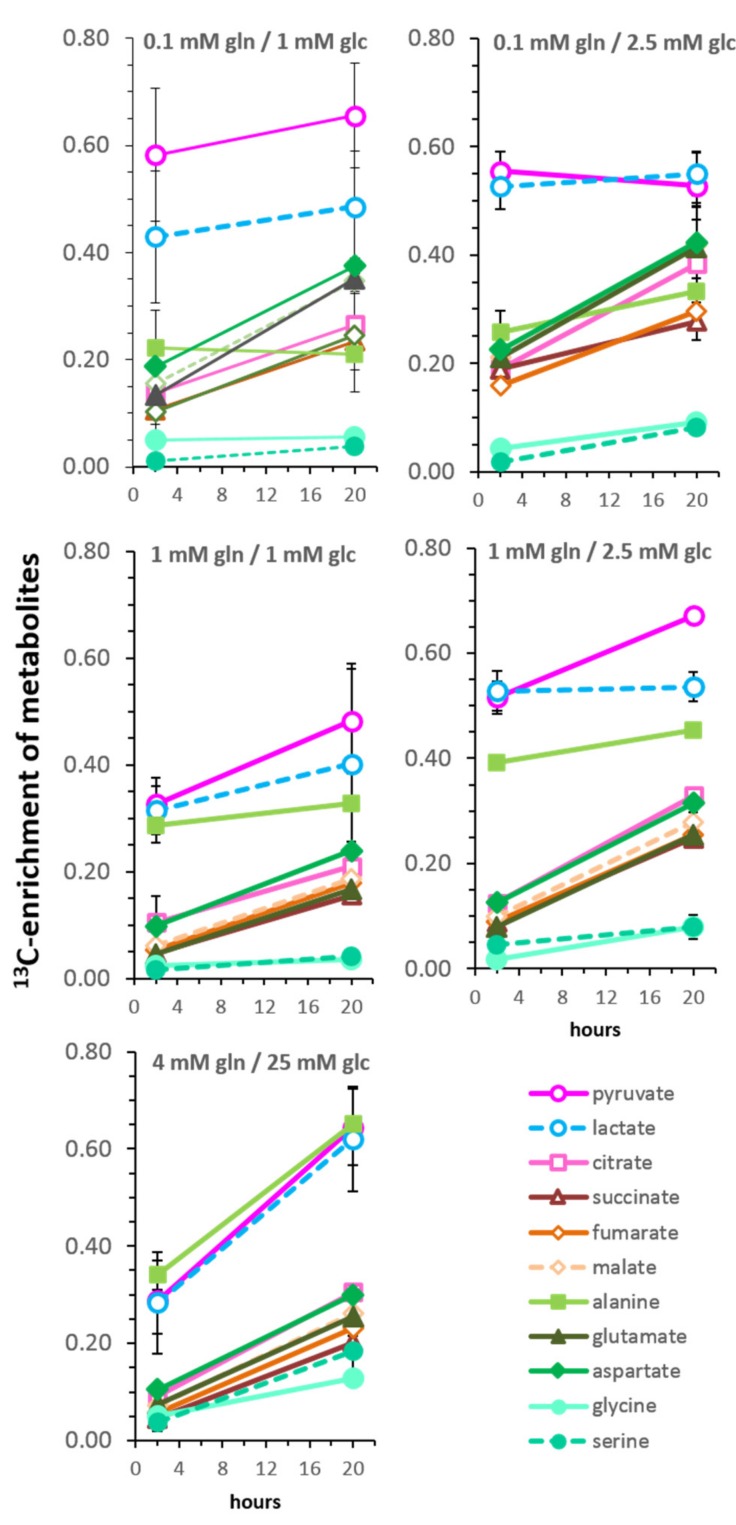
Time-dependent changes in the ^13^C-enrichment of metabolites. Shown are the values following incubations for 2 h (Figure 3) and 20 h (Figure S4) with [U-^13^C_6_]glucose in different glucose and glutamine concentrations as indicated in each graph. Amino acids are depicted in green colors.

Comparing the ^13^C-enrichment of metabolites at 2 and 20 h of incubation with [U-^13^C_6_]glucose provides a qualitative estimation of the metabolic flux (Figure 5). For most metabolites, the highest rate of ^13^C-labeling occurred within the first 2 h after medium replenishment. It should be noted, however, that by 20 h, the medium composition had changed, in particular in limiting glucose conditions, where the level (Figure 1B) and the rate of glucose uptake (Figure S1) declined. Nevertheless, ^13^C-metabolites can be grouped according to their relative enrichment values and their slopes. The group with the highest level of ^13^C-enrichement included pyruvate and lactate, while another group consisted of TCA-related metabolites including citrate, glutamate, succinate, fumarate, malate, and aspartate. Interestingly, the increase of the TCA-metabolites showed similar slopes of increase in all conditions. Such a grouping supports the concept of common metabolic pools or reaction compartments [33].

In saturating conditions, the metabolic ^13^C-labeling of serine and glycine occurred at a similar level and rate as the TCA-cycle related metabolites, even though their biosynthetic precursor 3-phosphoglycerate, is a metabolite of the glycolytic pathway. This is in contrast to the limiting glucose/glutamine conditions, where serine and glycine had a very low (<10%) level of ^13^C-enrichment and appeared as metabolites kinetically apart from the TCA-cycle metabolites. The metabolite with the highest variability between the different growth conditions was ^13^C-alanine; in saturating conditions, it grouped with pyruvate and lactate. However, in limiting glucose conditions with 0.1 mM glutamine, ^13^C-alanine enrichment was similar to that of TCA cycle metabolites, albeit with no or little increase at 20 h. However, in conditions with 1 mM glutamine, ^13^C-enrichment of alanine was positioned between the glycolytic and TCA cycle-related metabolites, suggesting alternating routes for alanine biosynthesis depending on the glutamine levels.

### 3.6. Isotopologue Profiles and Plasticity of Engaged Metabolic Pathways

#### 3.6.1. ^13^C-Metabolites Related to Glycolysis

**Figure 6:**
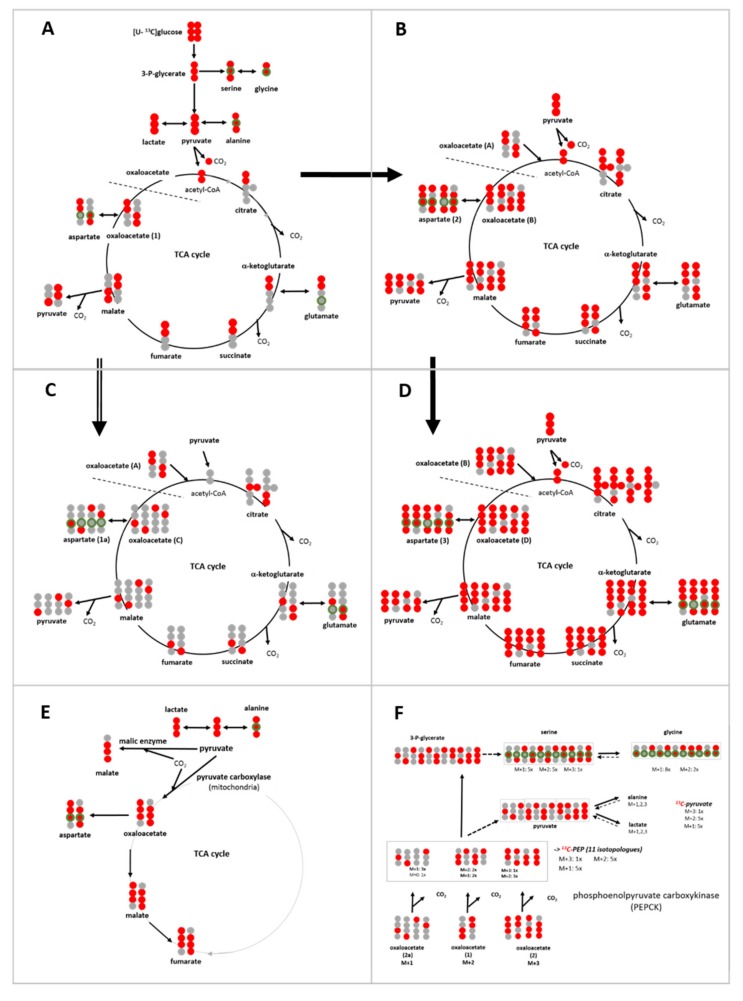
Schemes of isotopologues of ^13^C-labeled metabolites evolving in different metabolic pathways following uptake of [U-^13^C_6_]glucose; (**A**) glycolysis and *first* round of the TCA cycle. (**B**) *Second* round of the TCA cycle with ^13^C_2_-acetyl-CoA and ^13^C-oxaloacetate from the *first* TCA cycle. (**C**) *Second* round of the TCA cycle with unlabeled acetyl-CoA and ^13^C-oxaloacetate from the *first* TCA cycle, leading to M+1 metabolites. (**D**) *Third* round of the TCA cycle with ^13^C_2_-acetyl-CoA and ^13^C-oxaloacetate from the *second* round of the TCA cycle. (**E**) Isotopologues of TCA-cycle metabolites resulting from pyruvate anaplerosis via pyruvate carboxylation. (**F**) Possible ^13^C-isotopologues derived from ^13^C-oxaloacetate, resulting from different rounds of the TCA cycle, and used for gluconeogenesis. The expected numbers of isotopologues were calculated assuming for simplicity that each ^13^C-oxaloacetate pool contributed equally. ^13^C-atoms are indicated by red filled circles; green circles indicate amino sites.

**Figure 7:**
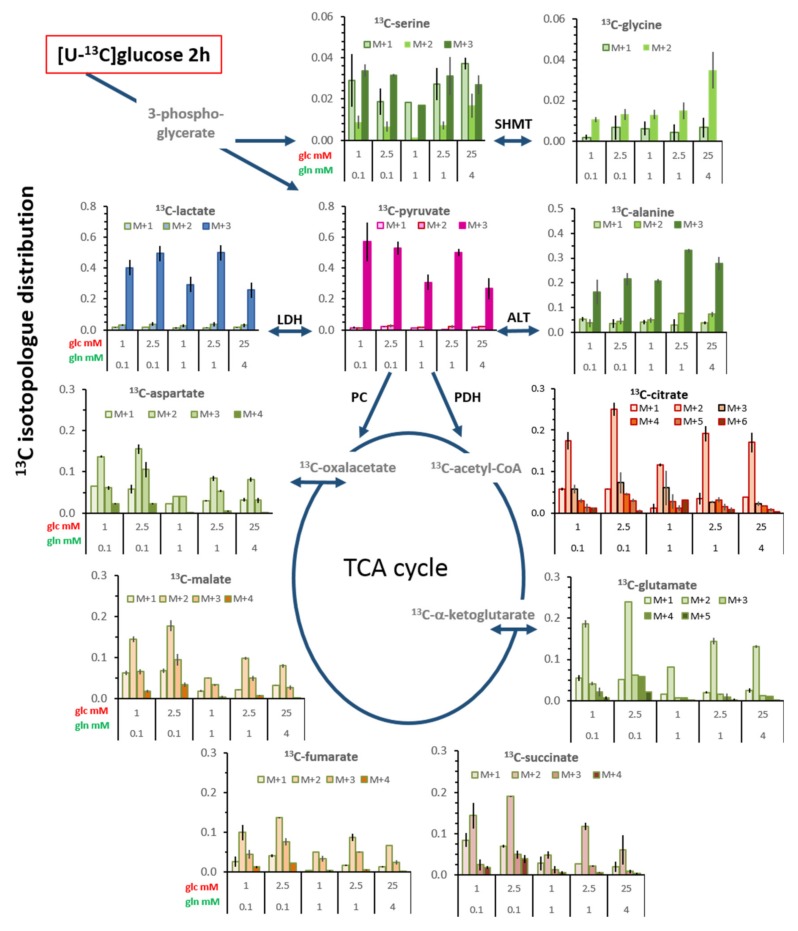
Isotopologue profiles of ^13^C-labeled metabolites associated with glycolysis and the TCA-cycle after a 2 h incubation with [U-^13^C_6_]glucose. The isotopologue fractions were calculated for the total metabolite pools (including M+0) (Figure S7B). The isotopologue profiles for cells after a 20 h incubation are shown in Figure S10. Each bar shows the average and range of values from individual experiments. Also, the isotopologue distribution of only the ^13^C-labeled metabolites (Figure S7A) at 2 and 20 h are presented in the Supplementary Figures S7 and S8.

Upon incubation with [U-^13^C_6_]glucose, the metabolites related to the glycolytic pathway are expected to be initially uniformly ^13^C-labeled (Figure 6A), and this applied to about 95% of ^13^C-pyruvate and >85% of ^13^C-lactate at 2 h (Supplementary Figure S8). After 20 h (Supplementary Figure S9), these values for M+3 pyruvate decreased to about 85%, mainly in conditions with 1 mM glutamine, with a variable increase in M+2 and M+1. In comparison, ^13^C-lactate had an increase mainly in the M+2 fraction. This altered profile indicates metabolic diversions of pyruvate carbons, probably through the TCA-cycle via malate; formally, replenishment from the gluconeogenic pathway from oxaloacetate could also be possible (Figure 6). Interestingly, for these *de novo* metabolites, at 2 h, the largest fractions of M+2 and M+1 pyruvate and lactate were observed in standard conditions, suggesting that pyruvate replenishment is not confined to limiting glucose and glutamine conditions. When considering the levels of M+3 isotopologues in the total pyruvate and lactate pools (Supplementary Figure S7B), these amount up to about 60% and 40%, respectively, having been newly synthesized via glycolysis (Figure 7).

Of the ^13^C-serine pool, 40%–50% was fully labeled and 70%–80% of glycine (M+2) (after 2 h, Figure S8), likewise suggesting a role of gluconeogenesis following transfer of the glucose carbons through the TCA-cycle (Figure 6). Again, standard conditions resulted in a higher fraction of M+2 and M+1 than the limiting conditions, this fraction being even increased after 20 h (Figure S8). However, as extracellular sources can maintain the intracellular levels of serine and glycine (Figure 2) [34], *de novo* synthesis from the glycolytic intermediate contributed only about 3% and 1%, respectively, to their total pools (Figure 7).

**Figure 8:**
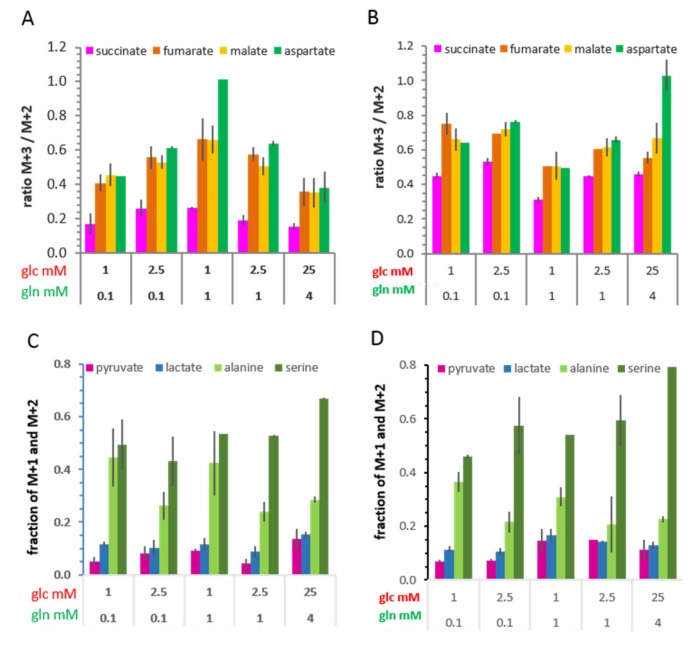
Isotopologue distributions providing evidence for pyruvate anaplerosis (**A**,**B**) and for replenished pyruvate and gluconeogenesis (**C**,**D**) in different glucose/glutamine conditions. Increased M+3/M+2 ratios for fumarate, malate, and aspartate (proxy for oxaloacetate) compared with succinate are indicative of pyruvate anaplerosis (Figure 6E), (A) at 2 h and (B) at 20 h of incubation with [U-^13^C_6_]glucose. On the other hand, the contributions of M+1 and M+2 to the ^13^C-isotopologue profiles of pyruvate, lactate, and alanine indicate replenishment of pyruvate from ^13^C-malate (Figure 6A–D) and possibly gluconeogenesis (Figure 6F), while the isotopologue profiles of serine could indicate gluconeogenesis from ^13^C-oxaloacetate at (C) 2 h and (D) 20 h. Each bar shows the average and range of values from individual experiments.

In contrast to lactate, ^13^C-alanine, as the transamination product of ^13^C-pyruvate, was only 60%–70% fully labeled at 2 h (Supplementary Figure S8), and similarly at 20 h (Supplementary Figure S9), amounting to only 20–30% being *de novo* in the total pool (Figure 7). Moreover, the combined fractions of M+1 and M+2 ^13^C-alanine were much higher than those of ^13^C-pyruvate, especially in the conditions with 1 mM glucose, at both 2 h and 20 h (Figure 8A,B). These M+2 and M+1 isotopologues suggest that metabolically replenished ^13^C-pyruvate was a preferred substrate for transamination to alanine.

#### 3.6.2. ^13^C-Metabolites Related to the TCA cycle and Pyruvate Anaplerosis

The ^13^C-isotopologue profiles of metabolites of the TCA cycle after a 2 h incubation also showed glucose/glutamine differences (Figure 7, Figure S8). The most prominent isotopologue species was M+2, resulting from a first canonical round of the TCA cycle (Figure 6A); subsequent canonical rounds of the TCA cycle led either to M+1 or to M+3 and M+>4 isotopologues (Figure 6B–D). The M+2 fraction of each metabolite was highest in the condition with 2.5 mM glucose/0.1 mM glutamine, and lowest with 1 mM glucose/1 mM glutamine, underlining the differences between these nutrient conditions. Moreover, the fraction of ^13^C-glutamate isotopologues with M+4 and M+5 was lower in conditions with 1 and 4 mM glutamine than the limiting 0.1 mM glutamine, a further indication for glutamine anaplerosis at both time points (Figure 7; Supplementary Figure S10). Notably, at 2 h, the fraction of M+3 isotopologues of ^13^C-fumarate, ^13^C-malate, and ^13^C-aspartate was about two-fold higher than those of the preceding metabolite ^13^C-succinate at 2 h (Figure 7 and Figure 8A). This can be explained by M+3 ^13^C-pyruvate entering the TCA cycle via the pyruvate carboxylase reaction, that is, pyruvate anaplerosis (Figure 6E). As the fraction of M+2 remained higher than that of M+3 for these metabolites, pyruvate anaplerosis made a subordinate contribution to the TCA cycle. After 20 h, the ratio of M+3/M+2 markedly increased, especially for succinate, along with further increases in both M+3 and ≥M+4 isotopologues of the other metabolites (Figure 8B, Supplementary Figure S10), which is an indication for further rounds of the oxidative TCA cycle. Nevertheless, pyruvate anaplerosis occurred in all growth conditions, being higher in limiting than in saturating standard conditions.

#### 3.6.3. ^13^C-Metabolites Related to Gluconeogenesis

Evidence for the utilization of this pathway is provided by the fraction of M+1 and M+2 isotopologues of the glycolytic metabolites pyruvate and lactate (Figure 6F). As mentioned above, ^13^C-serine also consisted of a >50% fraction of M+2 and M+1 (Figure 8C,D), which could suggest biosynthesis from gluconeogenic isotopologues of 3-phosphoglycerate (Figure 6F). The fact that the fraction of M+1 ^13^C-serine was almost as high as that of its M+3 isotopologues could suggest that gluconeogenesis is initiated with M+1 oxaloacetate foremost in limiting glucose conditions. However, as the same isotopologue profiles were observed with saturating glucose conditions, gluconeogenesis appears not to be strictly an issue of extracellular glucose availability, concordant with it having a more comprehensive role in metabolism [25,35,36]. Alternatively, ^13^C-serine could also evolve from ^13^C-glycine via the reversible serine hydroxymethyl transferase (SHMT) reaction; however, this reaction does not easily explain the high fraction of M+1 serine compared with its M+2 isotopologue. Discerning the contribution of these different metabolic pathways will require further analysis.

## 4. Discussion

The central question of this study was how limiting nutrient levels of a cancer microenvironment alters glucose-derived metabolism in tumor cells. Our results based on the enrichment and isotopologue profiles of ^13^C-glucose-derived metabolites show that in variable limiting glutamine and glucose conditions, the same metabolic roads are travelled by, but with changes in direction and metabolic traffic. This dexterity allows for rapid metabolic plasticity without requiring changes in the expression of metabolic enzymes.

One level of metabolic plasticity is the variability in the content of metabolites. The relative quantification of pyruvate by GC/MS suggests that pyruvate, but also citrate, maintains a small pool size with little variation in the different glucose/glutamine conditions (Figure 2). This is concordant with a central metabolic role, which could be envisaged as a revolving door of limited substrate capacity controlling the entry into subsequent metabolic reaction compartments. The intracellular concentrations of lactate, however, remained comparatively constant (at about 2 mM) over a range of low glucose/glutamine conditions and did not correlate with glucose consumption and lactate release. Increased intracellular lactate concentrations were observed particularly for standard conditions and the combination of 2.5 mM glucose/0.1 mM glutamine, which went along with increased pyruvate kinase and LDH activities. A large sustaining lactate pool may function as a flow-through reservoir, which, owing to the high conversion rate by LDH [32], could supply pyruvate virtually instantaneously. When the rate of glycolysis produces a surplus of lactate, this could be expelled by the cells. This constitutes the Warburg effect, which can be viewed as a corrective element of aberrant control of glycolysis. Inversely, excess lactate released could serve as a signaling molecule and feed other tumor as well as stroma cells, by providing a precursor for pyruvate and thus intermediate metabolites of glycolysis through gluconeogenesis, thereby supporting survival when glucose becomes limiting for proliferation [37,38].

Another level of metabolic plasticity is reflected in the metabolic flux. The ^13^C-enrichment and the isotopologue profiles of pyruvate and lactate indicate that these two metabolites are mainly direct products of glycolysis. Glycolysis is also the source for about 50% of serine biosynthesis (via 3-phosphoglycerate), as suggested by its isotopologue profile. However, the high fraction of serine isotopologues with reduced ^13^C-labeling (M+2, M+1) suggests that serine synthesis also utilizes precursors resulting from other metabolic pathways, one being the conversion of ^13^C-glycine by SHMT, both isotypes 1 and 2 being expressed in MCF-7 cells [39,40,41]. Alternatively, and/or additionally, serine could be the result of gluconeogenesis providing 3-phosphoglycerate, which better explains the high fraction of M+1 compared with M+2 isotopologues. (Figure 6). This latter hypothesis is supported by tracer studies performed with lung cancer cell lines [25]. Further evidence for the interplay of glycolysis and gluconeogenesis is provided by structural imaging at the cellular level, showing that glycolytic and gluconeogenic enzymes appear in common clusters [42]. This supports an integrative role of gluconeogenesis in metabolic programming, such as coordinating glucose and glutamine utilization, and entry of glutamine into the TCA cycle [36], as well as metabolic guidance of lactate and alanine to gluconeogenesis [25].

The metabolism of alanine (not provided in the medium) showed differential flexibility with respect to the origin of its precursor pyruvate. In the most limiting (1 mM) glucose conditions, the rate of ^13^C-alanine enrichment was similar to that of TCA cycle metabolites (Figure 5). The deviant isotopologue distributions of ^13^C-alanine compared with those of ^13^C-lactate (Figure 7 and Figure 8A,B) also suggest the use of different pyruvate sources for transamination; while the M+3 isotopologues are derived directly from glycolysis, the M+1 and M+2 pyruvate are likely to be the result of decarboxylation of ^13^C-malate from the TCA cycle (although gluconeogenic pyruvate cannot be excluded) (Figure 6). The fraction of ^13^C-alanine derived from replenished pyruvate was rather high, about 40% in limiting (1 mM) glucose conditions. Our results thus show that reduced glucose availability shifted the metabolic route for alanine transamination from glycolytic to replenished pyruvate, which is concordant with the existence of a cytosolic pyruvate compartment favoring alanine conversion [43], and which may reflect differences in cytosolic and mitochondrial sites and the activities of alanine transaminase ALT1 and ALT2 [44].

The metabolic contribution of extracellular glutamine is tightly affiliated with the concentrations of available glucose [17], as also shown by other studies, albeit generally with saturating glutamine in combination with glucose deprivation [15,45,46]. We show here that intracellular glutamate levels are related to the levels of extracellular glutamine in combination with available glucose (Figure 2). Moreover, the isotopologue profiles of ^13^C-glutamate and of ^13^C-fumarate, ^13^C-malate, and ^13^C-aspartate (proxy for oxaloacetate) (Figure 7) reflect this metabolic coupling of glucose and glutamine; in conditions with saturating glutamine, the anaplerotic contribution of glutamine to the TCA cycle is manifested by an about 50% reduction of ^13^C-enrichment of these metabolites. Also, the enrichment of ^13^C-citrate was reduced, which indicates reductive carboxylation of α-ketoglutarate contributed by extracellular glutamine [47]. Another study showing the labelling of α-ketoglutarate upon incubation with ^13^C-glutamine in MCF-7 cells corroborates this conclusion [48]. However, glutamine levels did not affect pyruvate carboxylation to oxaloacetate, as indicated by its indifference to glutamine levels (Figure 8C,D), suggesting that pyruvate and glutamine anaplerosis operate independently. This is in line with evidence that pyruvate anaplerosis could maintain tumor cell growth when extracellular glutamine was lacking [49].

A number of studies with different cancer cell types, including melanoma and different breast cancer cell lines [50,51,52], as well as our yet unpublished data with MDA-MB231 cells with limiting conditions, indicate that the same limiting nutrient environment can lead to different metabolic phenotypes. Our study illustrates the metabolic plasticity of cells both at the level of metabolites and the metabolic fates of ^13^C-glucose, reflecting the flexibility of anabolic and catabolic pathways in response to fluctuating nutrient conditions. To better mimic the tumor tissue in vivo, further studies will need to include other parameters of the microenvironment, for example, acidic pH and hypoxia. Thereby, this cell culture system, even though seemingly simplistic, can model different specific metabolic conditions, their contribution being blurred when assaying a heterogeneous tumor tissue on the whole, but which may be important for the further development of tumor growth or chemosensitivity. This line of thinking has just been confirmed in a preclinical model with a new glucose-imaging methodology showing differences in metabolism within the tumor [53]. Thus, our results underscore the importance of using tumor-relevant nutrient conditions for cell culture-based studies in providing more physiologically relevant information, especially when designing assays and protocols for the evaluation of drug screening, for the prediction of chemotherapeutic effects, as well as for metabolic imaging in the clinic.

## Supplementary Materials

The following are available online at https://www.mdpi.com/2073-4409/8/10/1113/s1, Gkiouli et al. Supplementary Information 13C-metabolites AO 190919.
