# Supplementary material for: Diverse Roads Taken by 13C-Glucose-Derived Metabolites in Breast Cancer Cells Exposed to Limiting Glucose and Glutamine Conditions
Source: Cells. 2019 Sep 20;8(10):1113. doi: 10.3390/cells8101113 (PMC6829299; doi:10.3390/cells8101113)
Supplement: Supplementary file 1 [file cells-08-01113-s001.pdf]

Supplementary Information

# Diverse roads taken by $^{13}$C-glucose-derived metabolites in tumor cells exposed to limiting glucose and glutamine conditions

Maria Gkiouli, Philipp Biechl, Wolfgang Eisenreich,

Angela M. Otto (*otto@tum.de)*

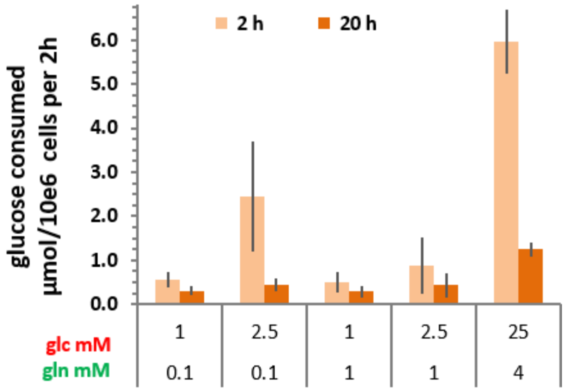


**Figure S1: Cellular glucose consumption rate** at 2h and 20 h of incubation after medium replenishment with the indicated glucose / glutamine conditions. Cells were cultured in 6-well plates as described in Material and Methods. Consumption was calculated from the amount of glucose removed from the supernatant (Fig. 1B), i.e. the difference to the initial concentration in the medium, and related to the number of cells in the culture. The value of consumed glucose at 20 h was normalized to a 2 h increment. The graph shows that glucose consumption rate decreased with incubation time.

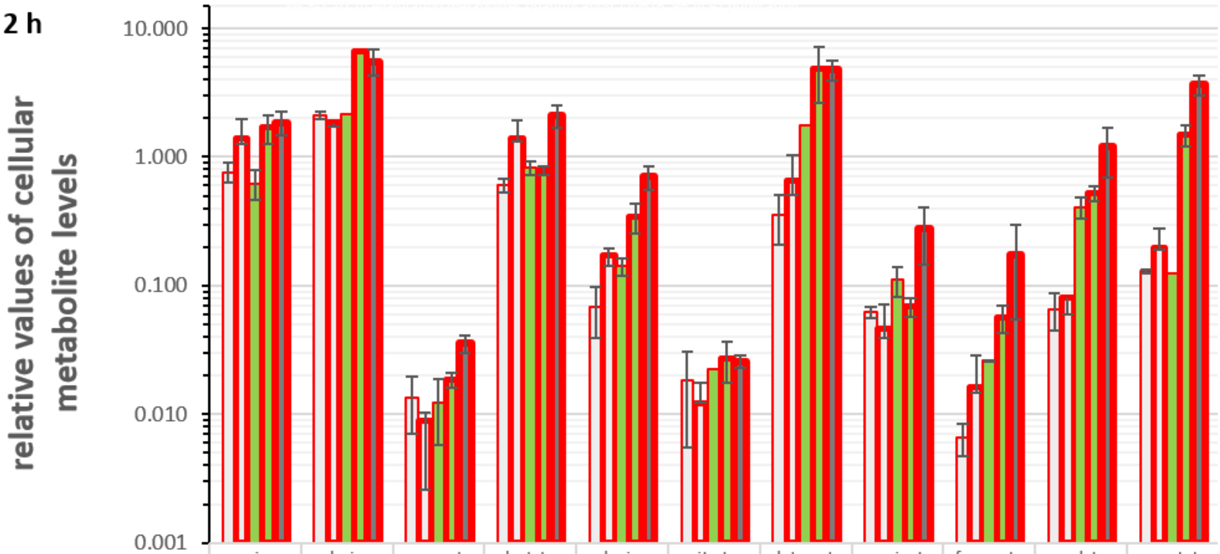


| | serine | glycine | pyruvate | lactate | alanine | citrate | glutamate | succinate | fumarate | malate | aspartate |
|---|---|---|---|---|---|---|---|---|---|---|---|
| 1 mM glc / 0.1 mM gln | 0.763 | 2.116 | 0.013 | 0.605 | 0.068 | 0.018 | 0.357 | 0.062 | 0.007 | 0.065 | 0.129 |
| 2.5 mM glc / 0.1 mM gln | 1.394 | 1.855 | 0.009 | 1.396 | 0.170 | 0.012 | 0.659 | 0.046 | 0.016 | 0.080 | 0.195 |
| 1 mM glc / 1 mM gln | 0.623 | 2.171 | 0.012 | 0.820 | 0.142 | 0.022 | 1.765 | 0.111 | 0.026 | 0.410 | 0.123 |
| 2.5 mM glc / 1 mM gln | 1.699 | 6.678 | 0.018 | 0.780 | 0.347 | 0.027 | 4.875 | 0.068 | 0.057 | 0.525 | 1.490 |
| 25 mM glc / 4 mM gln | 1.856 | 5.541 | 0.036 | 2.100 | 0.704 | 0.026 | 4.803 | 0.276 | 0.175 | 1.199 | 3.642 |

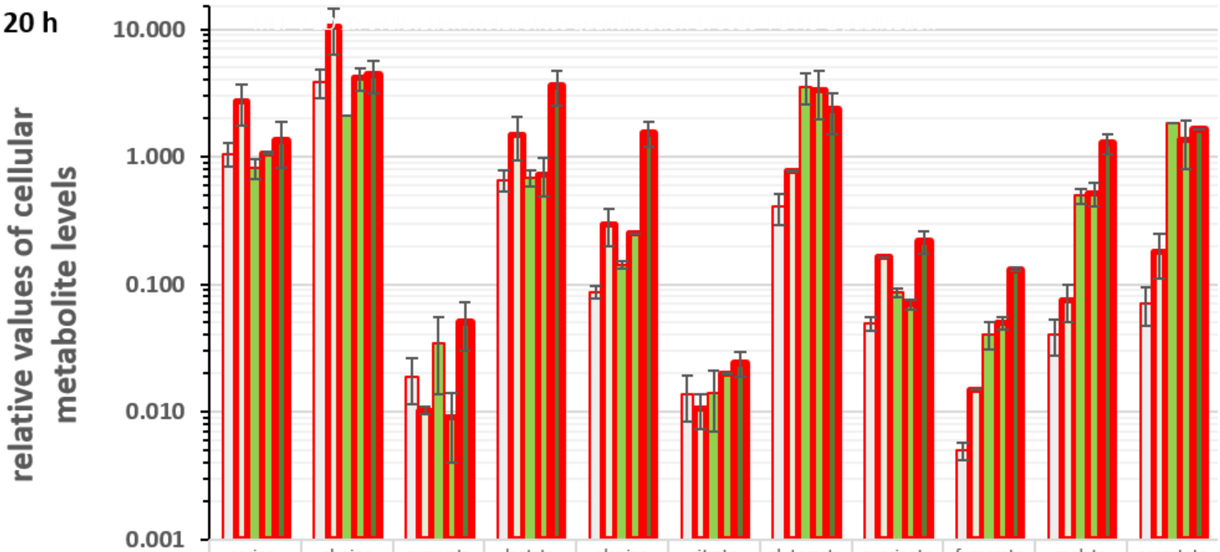


| | serine | glycine | pyruvate | lactate | alanine | citrate | glutamate | succinate | fumarate | malate | aspartate |
|---|---|---|---|---|---|---|---|---|---|---|---|
| 1 mM glc / 0.1 mM gln | 1.056 | 3.869 | 0.0189 | 0.6557 | 0.0871 | 0.0138 | 0.4038 | 0.0494 | 0.0049 | 0.0404 | 0.0707 |
| 2.5 mM glc / 0.1 mM gln | 2.722 | 10.397 | 0.0102 | 1.4997 | 0.2941 | 0.0106 | 0.7725 | 0.1635 | 0.0149 | 0.0748 | 0.1795 |
| 1 mM glc / 1 mM gln | 0.816 | 2.107 | 0.0345 | 0.6822 | 0.1413 | 0.0140 | 3.5324 | 0.0861 | 0.0405 | 0.4958 | 1.8479 |
| 2.5 mM glc / 1 mM gln | 1.069 | 4.133 | 0.0090 | 0.7304 | 0.2501 | 0.0197 | 3.3198 | 0.0694 | 0.0493 | 0.5181 | 1.3639 |
| 25 mM glc / 4 mM gln | 1.355 | 4.429 | 0.0507 | 3.5898 | 1.5397 | 0.0243 | 2.3391 | 0.2151 | 0.1309 | 1.2747 | 1.6380 |

**Figure S2: Relative cellular levels of metabolites** detectable at 2 hr and 20 hr after medium change with the indicated $^{13}$C-glucose / glutamine concentrations. The metabolite peaks from GC-MS analysis were quantified relative to 0.4. mM norvaline (=1) added as a reference compound. The relative quantities are depicted in a logarithmic scale. The corresponding graphs at 2 and 20h are depicted in Fig 2 and Fig. S3.

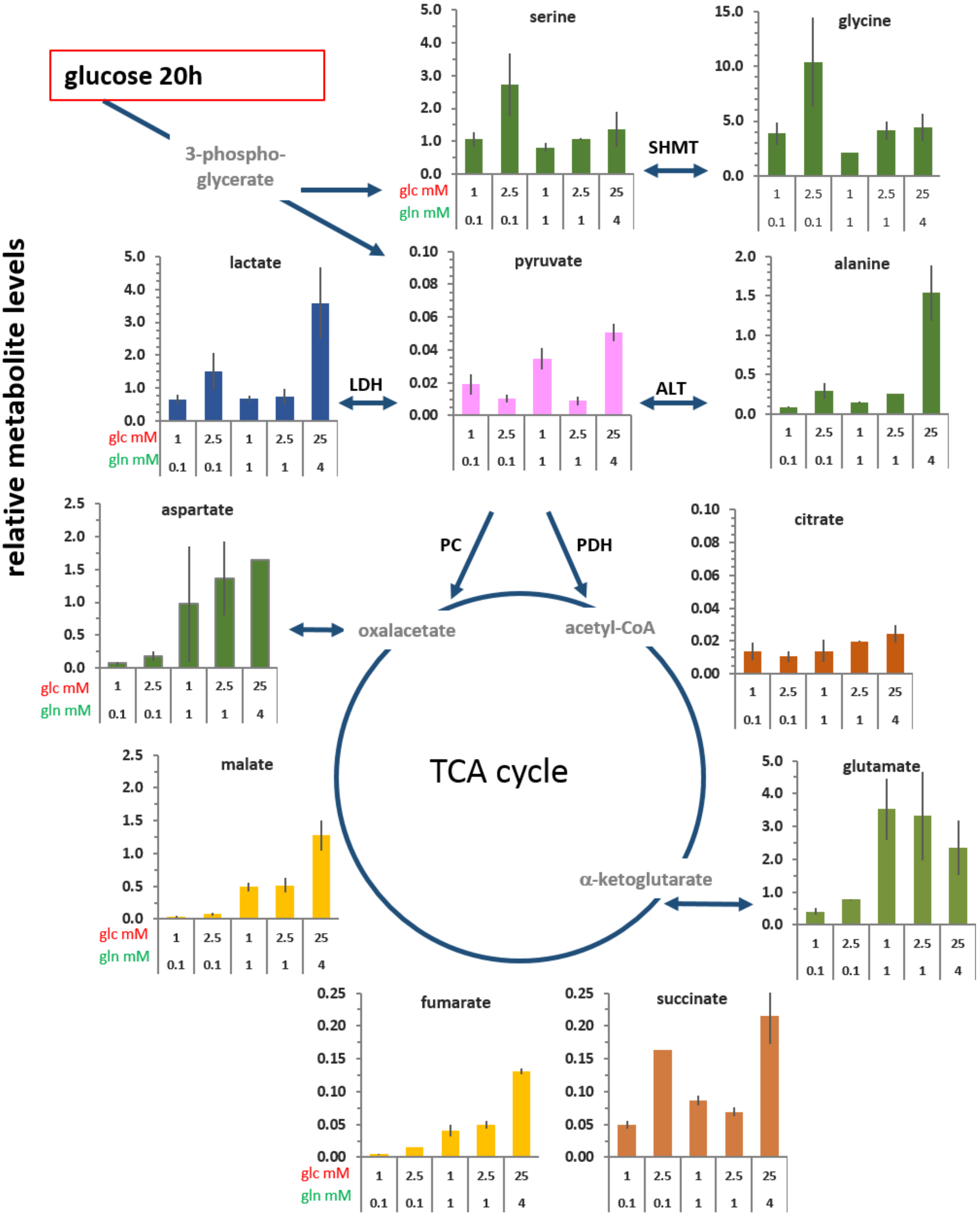


**Figure S3: Cellular metabolite levels 20 h after medium replenishment with various glucose / glutamine combinations**. The metabolites detected by GC-MS were quantified with reference to 0.4 mM norvaline (=1). The data of these metabolites for cells at 2 and 20 h are compiled in Fig. S2. SHMT: serine hydroxymethyl transferase; LDH: lactate dehydrogenase; ALT: alanine aminotransferase; PC: pyruvate carboxylase; PDH: pyruvate dehydrogenase. Oxaloacetate, acetyl-CoA and α-ketoglutarate were below the detection limit.

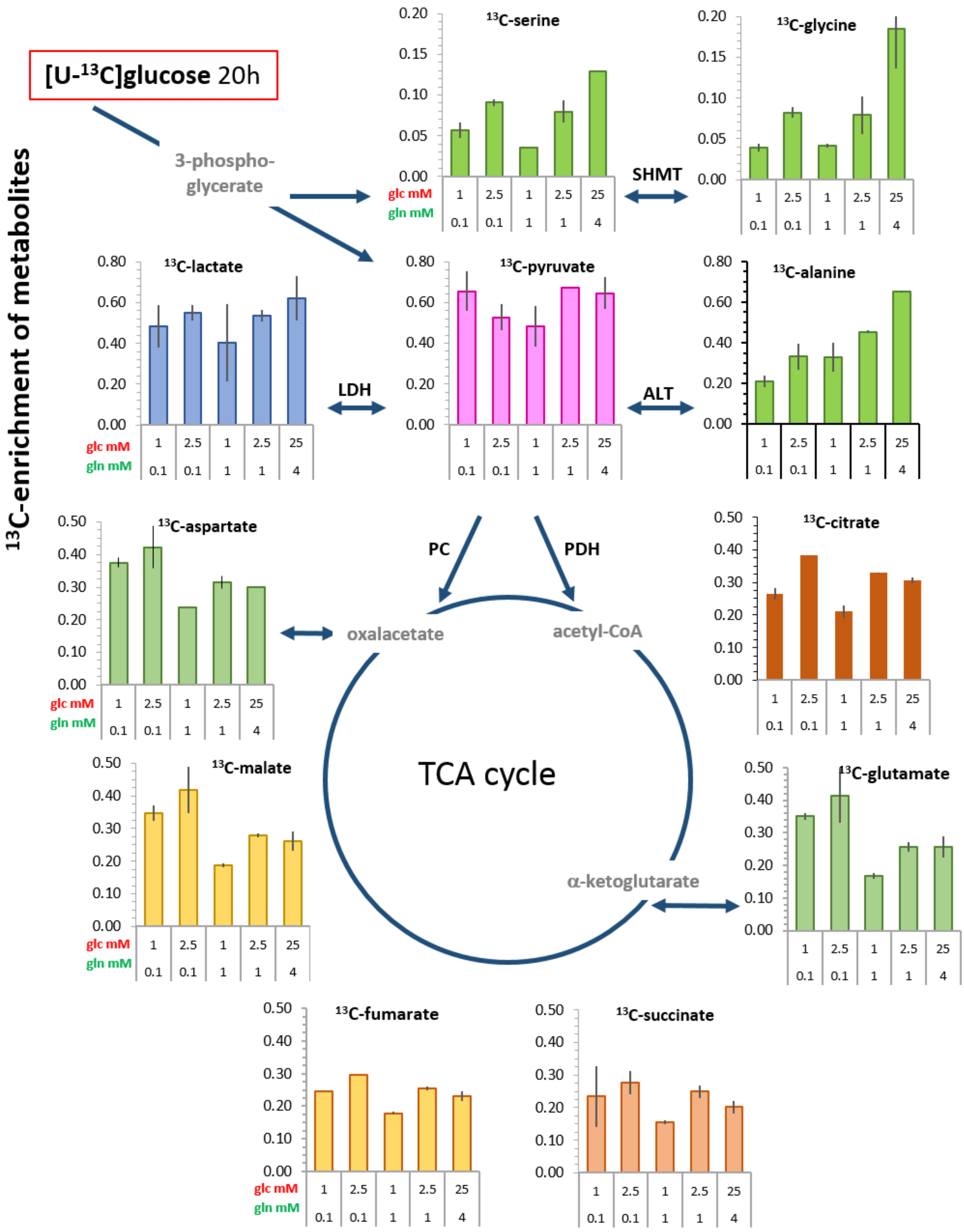


**Figure S4: $^{13}$C-enrichment of metabolite pools in cells incubated for 20 h** with [U-$^{13}C_6$]glucose in growth conditions with different combinations of glucose and glutamine concentrations at the indicated concentrations. Abbreviations as in Fig. S3.

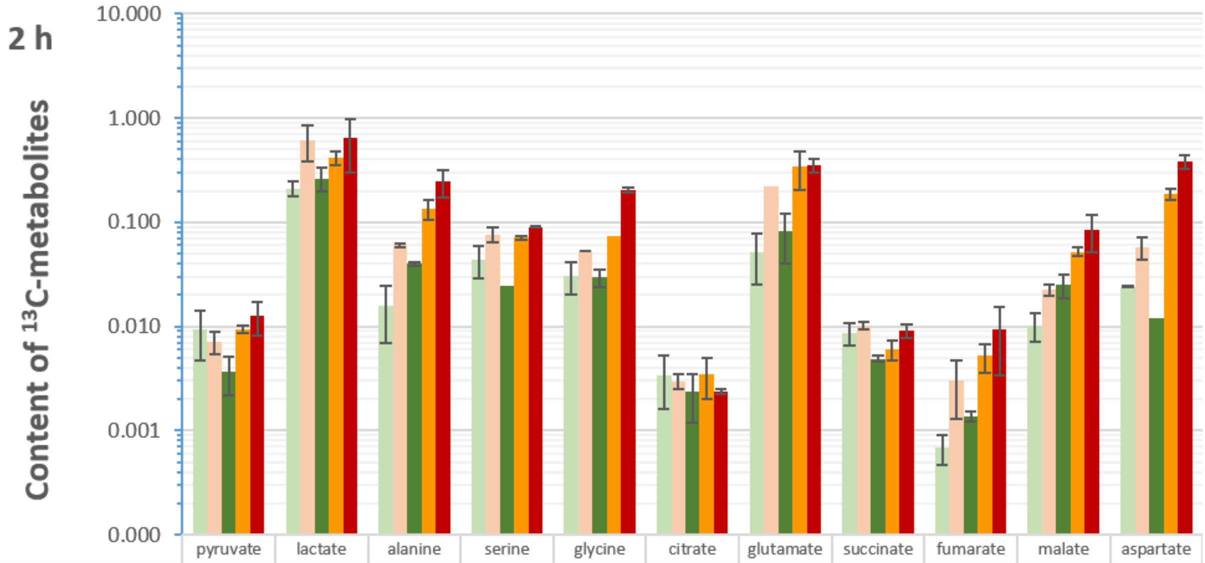


| | pyruvate | lactate | alanine | serine | glycine | citrate | glutamate | succinate | fumarate | malate | aspartate |
|---|---|---|---|---|---|---|---|---|---|---|---|
| 1 mM glc 0.1 mM gln | 0.009 | 0.212 | 0.016 | 0.044 | 0.031 | 0.003 | 0.051 | 0.009 | 0.001 | 0.010 | 0.024 |
| 2.5 mM glc 0.1 mM gln | 0.007 | 0.615 | 0.061 | 0.076 | 0.052 | 0.003 | 0.219 | 0.010 | 0.003 | 0.023 | 0.057 |
| 1 mM glc 1 mM gln | 0.004 | 0.263 | 0.040 | 0.024 | 0.030 | 0.002 | 0.081 | 0.005 | 0.001 | 0.025 | 0.012 |
| 2.5 mM glc 1 mM gln | 0.009 | 0.414 | 0.134 | 0.071 | 0.073 | 0.003 | 0.339 | 0.006 | 0.005 | 0.052 | 0.186 |
| 25 mM glc 4 mM gln | 0.013 | 0.638 | 0.244 | 0.090 | 0.204 | 0.002 | 0.355 | 0.009 | 0.009 | 0.084 | 0.380 |

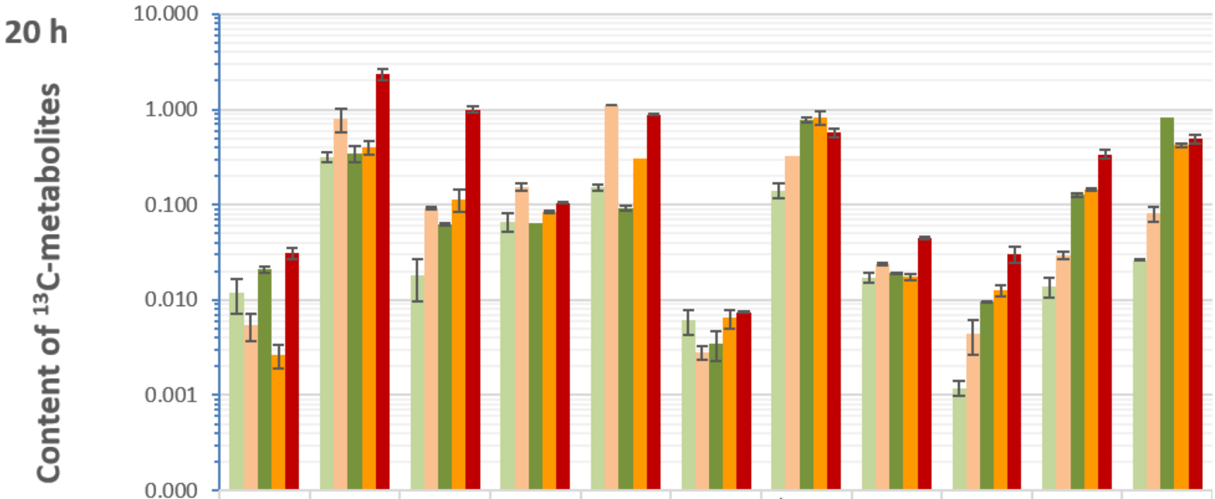


| | pyruvate | lactate | alanine | serine | glycine | citrate | glutamat e | succinate | fumarate | malate | aspartate |
|---|---|---|---|---|---|---|---|---|---|---|---|
| 1 mM glc 0.1 mM gln | 0.012 | 0.316 | 0.018 | 0.067 | 0.152 | 0.006 | 0.142 | 0.017 | 0.001 | 0.014 | 0.026 |
| 2.5 mM glc 0.1 mM gln | 0.005 | 0.802 | 0.092 | 0.153 | 1.097 | 0.003 | 0.321 | 0.024 | 0.004 | 0.030 | 0.080 |
| 1 mM glc 1 mM gln | 0.021 | 0.344 | 0.062 | 0.064 | 0.092 | 0.003 | 0.777 | 0.019 | 0.010 | 0.127 | 0.812 |
| 2.5 mM glc 1 mM gln | 0.003 | 0.398 | 0.113 | 0.085 | 0.307 | 0.006 | 0.829 | 0.017 | 0.013 | 0.144 | 0.420 |
| 25 mM glc 4 mM gln | 0.031 | 2.344 | 1.002 | 0.105 | 0.882 | 0.007 | 0.571 | 0.044 | 0.030 | 0.339 | 0.491 |

**Figure S5: Cellular quantification of the $^{13}$C-labelled metabolites** after 2 h and 20 -incubations of MCF-7 cells with [U-$^{13}C_6$]glucose. The $^{13}$C-enrichment value of each metabolite (Fig. S3) was multiplied with the total level of the metabolite determined (Fig. S2). The values give a semi-quantitative indication of the level of $^{13}$C-metabolites in the cells (see scheme in Fig. S7C). The corresponding graphs at 2 and 20h are depicted in Fig 4 and Fig. S6, respectively.

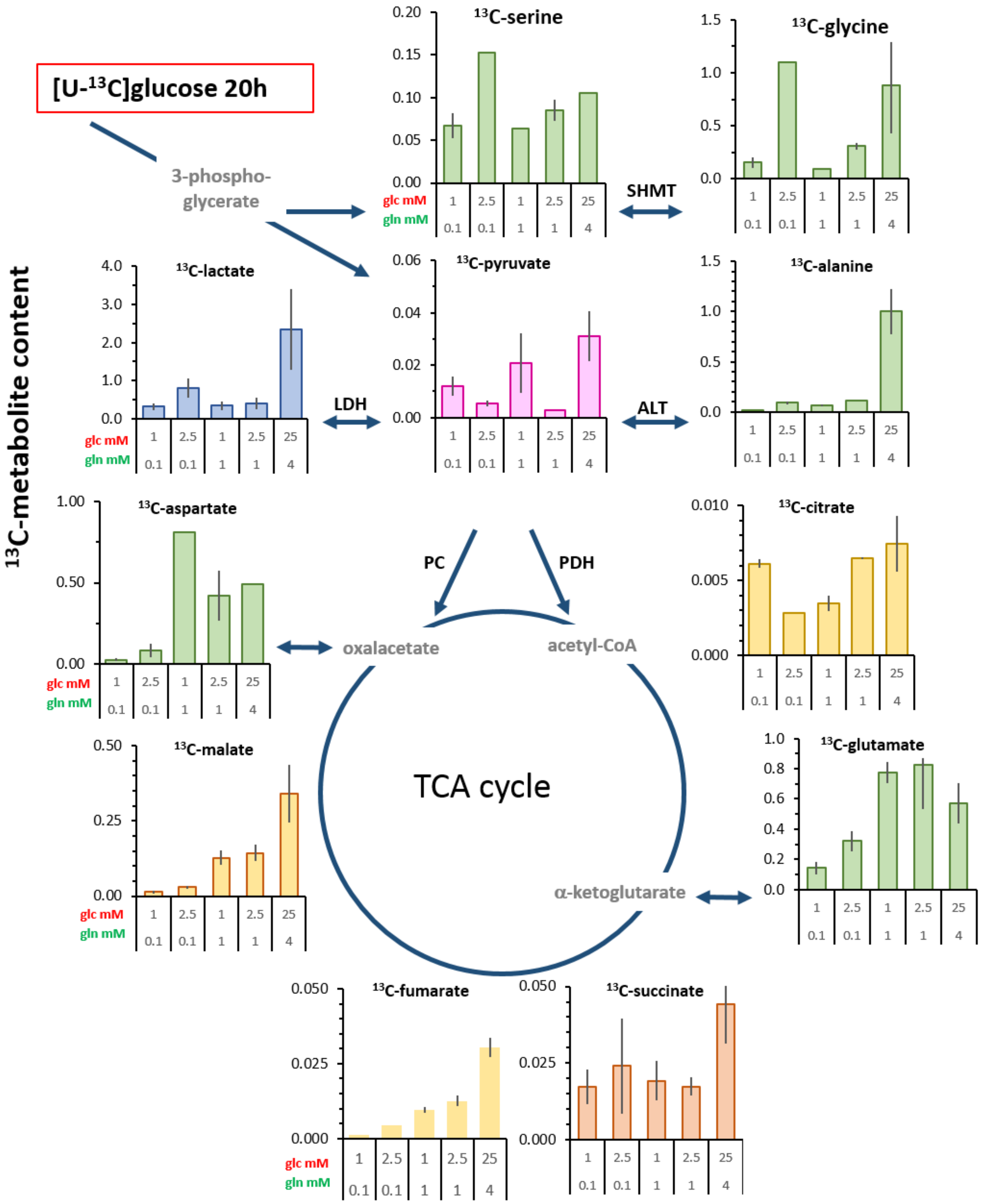


**Figure S6: Estimated cellular content of *de novo* glucose-derived metabolites** after a 20 h - incubation of MCF-7 cells with [U-$^{13}C_6$]glucose at the indicated glucose and glutamine concentrations. The values for each metabolite are provided in Fig. S5.

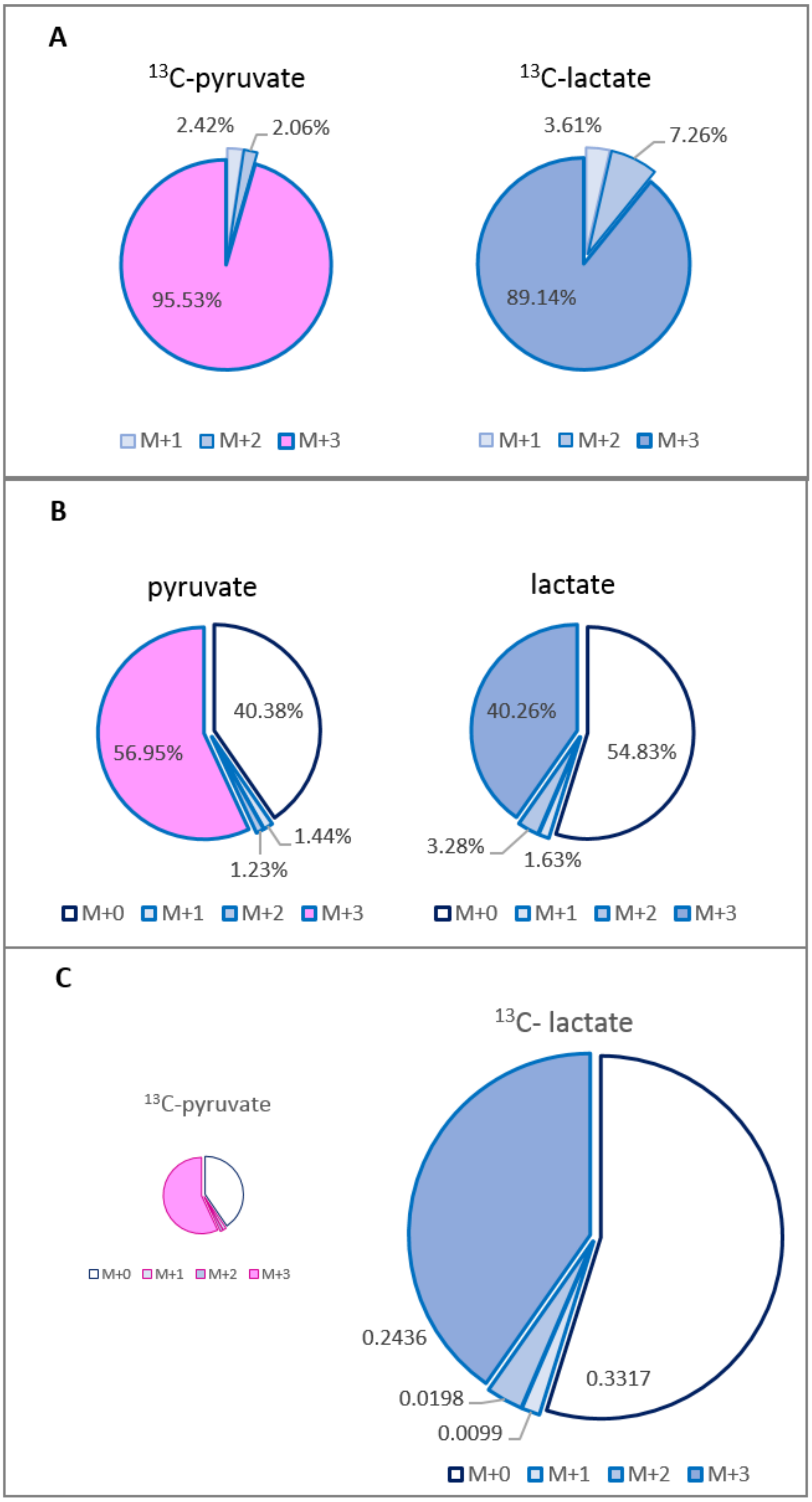


**Figure S7: Comparison of different $^{13}$C-isotopologue evaluations and their quantitative implications**. A) Fraction of different $^{13}$C-isotopologues within the $^{13}$C-labelled metabolite pool. B) Fraction of $^{13}$C- isotopologues within the total metabolite pool (incl. M+0 metabolites). C) $^{13}$C- isotopologues content taking in to account the cellular metabolite pool (diameter of the spheres are drawn approximately to scale).

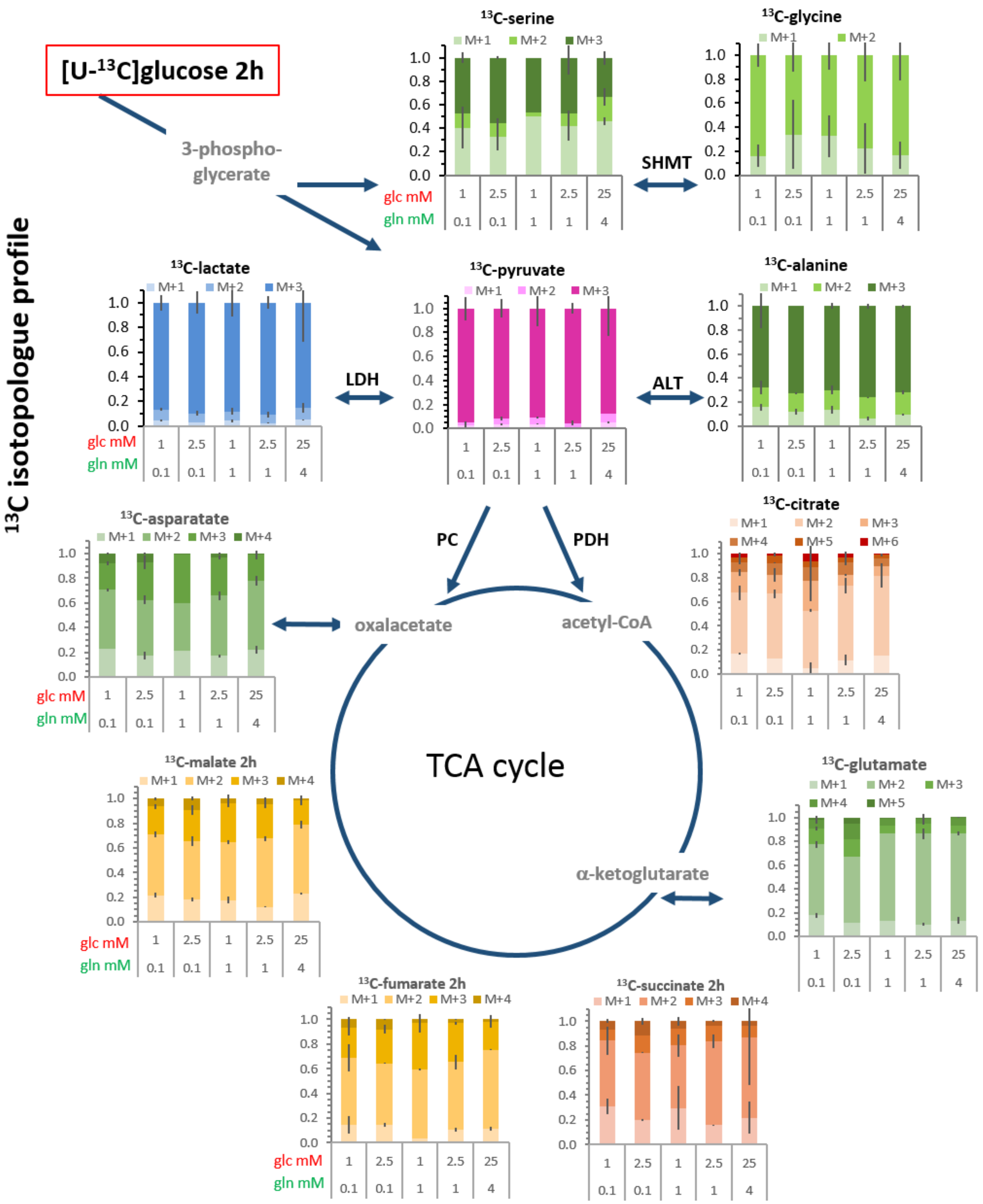


**Figure S8: $^{13}$C-isotopologue profiles of metabolites after 2 h** of [U-$^{13}C_6$]glucose incubation in different glucose / glutamine conditions. Shown are the mass distribution of the $^{13}$C-labelled metabolites evaluated as in Fig. S7A.

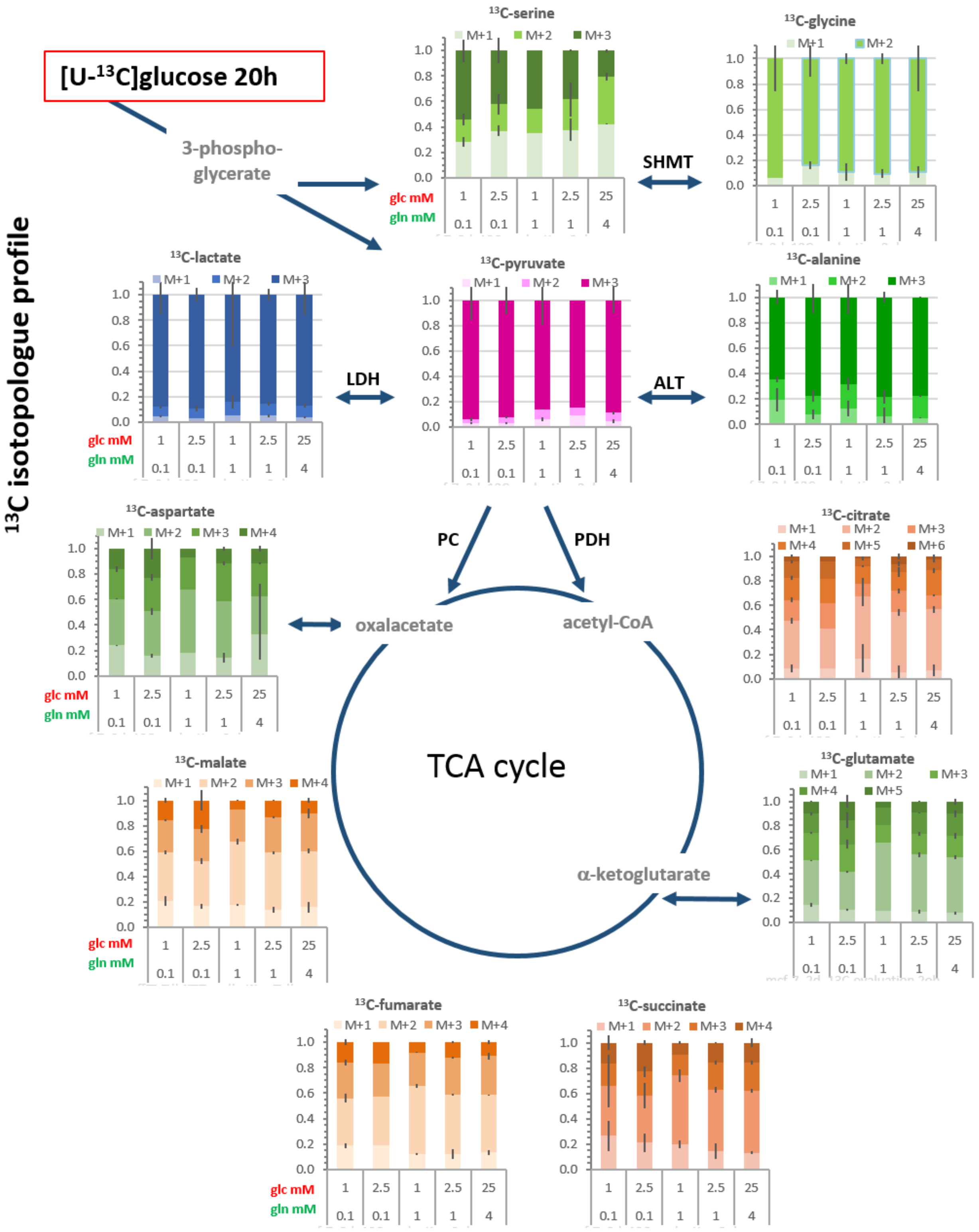


**Figure S9: 13C-isotopologue profiles of metabolites after 20 h** of [U-$^{13}C_6$]glucose incubation in different glucose / glutamine conditions. Shown are the mass distribution of the 13C-labelled metabolites evaluated as in Fig. S7A.

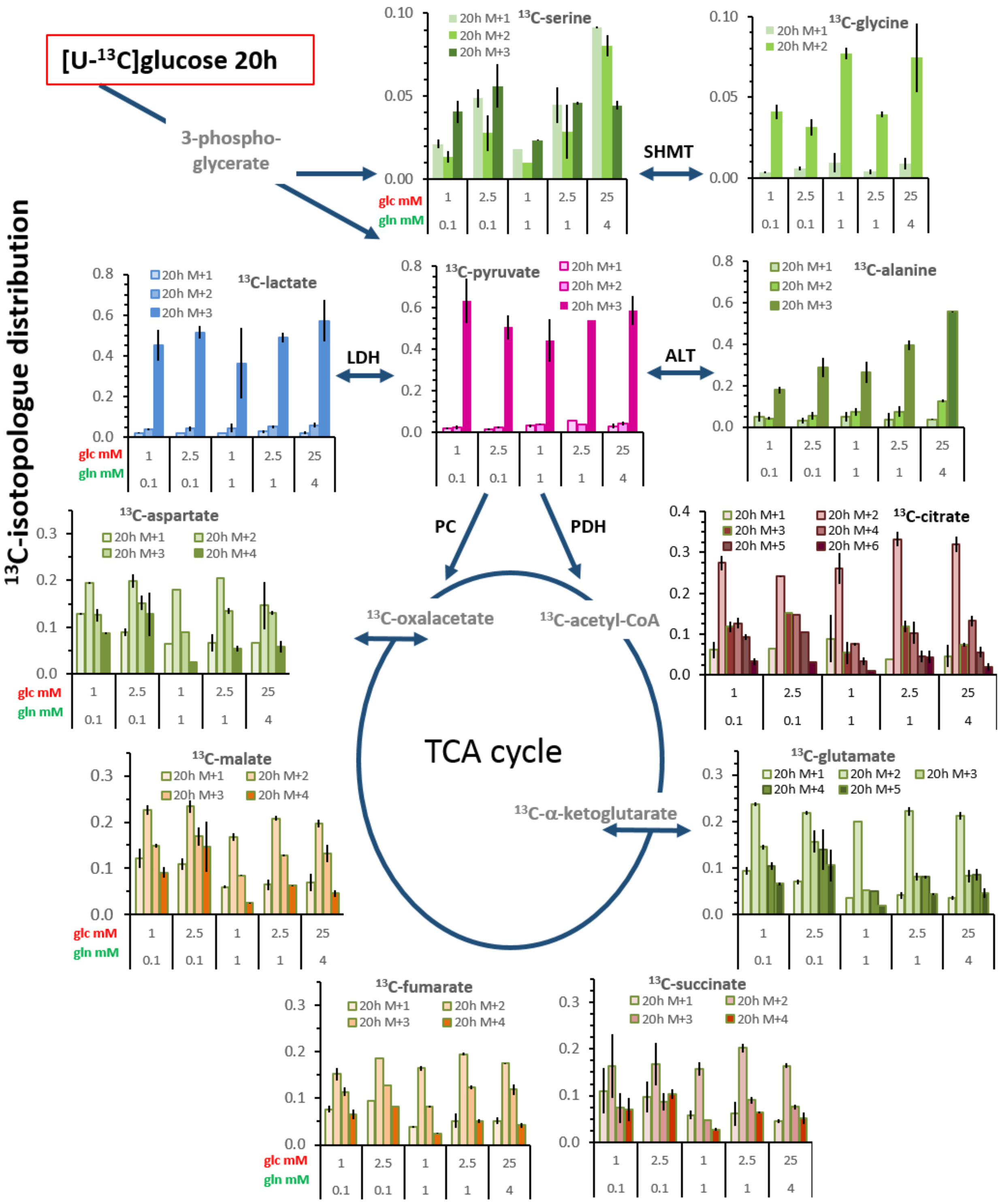


**Figure S10: Isotopologue profiles of $^{13}$C-labeled metabolites associated with glycolysis and the TCA-cycle after a 20 h-incubation** with [U-$^{13}C_6$]-glucose in different glucose /glutamine conditions. The isotopologue fraction was calculated from the respective total metabolite pools as depicted in Fig. S7B.
